# STIM2 regulates NMDA receptor endocytosis that is induced by short-term NMDA receptor overactivation in cortical neurons

**DOI:** 10.1007/s00018-023-05028-8

**Published:** 2023-11-21

**Authors:** Karolina Serwach, Ewa Nurowska, Marta Klukowska, Barbara Zablocka, Joanna Gruszczynska-Biegala

**Affiliations:** 1 Molecular Biology Unit, Mossakowski Medical Research Institute, Polish Academy of Sciences, Warsaw, Poland; 2 Department of Pharmacotherapy and Pharmaceutical Care, Centre for Preclinical Research and Technology (CePT), Medical University of Warsaw, Warsaw, Poland

**Keywords:** NMDA receptors, STIM proteins, Neuronal activation, Endocytosis, Internalization, Synaptosomes, Calcium, Whole-cell patch clamp, Cell surface, GluN2B, NMDAR overactivation, Lentiviruses, Receptor trafficking

## Abstract

Recent findings suggest an important role for the dysregulation of stromal interaction molecule (STIM) proteins, activators of store-operated Ca^2+^ channels, and the prolonged activation of *N*-methyl-D-aspartate receptors (NMDARs) in the development of neurodegenerative diseases. We previously demonstrated that STIM silencing increases Ca^2+^ influx through NMDAR and STIM–NMDAR2 complexes are present in neurons. However, the interplay between NMDAR subunits (GluN1, GluN2A, and GluN2B) and STIM1/STIM2 with regard to intracellular trafficking remains unknown. Here, we found that the activation of NMDAR endocytosis led to an increase in STIM2–GluN2A and STIM2–GluN2B interactions in primary cortical neurons. STIM1 appeared to migrate from synaptic to extrasynaptic sites. STIM2 silencing inhibited post-activation NMDAR translocation from the plasma membrane and synaptic spines and increased NMDAR currents. Our findings reveal a novel molecular mechanism by which STIM2 regulates NMDAR synaptic trafficking by promoting NMDAR endocytosis after receptor overactivation, which may suggest protection against excessive uncontrolled Ca^2+^ influx through NMDARs.

**Supplementary Information:**

The online version contains supplementary material available at 10.1007/s00018-023-05028-8.

## Introduction

Stromal interaction molecules (STIMs) are transmembrane proteins that are located mainly in the endoplasmic reticulum (ER), where they act as sensors of Ca^2+^ ion levels in both non-excitable [1, 2] and excitable cells (e.g., neurons; [3–8]. In the brain, STIM1 is found primarily in Purkinje neurons in the cerebellum, and STIM2 is found in the hippocampus and cortex [3, 9–11]. The main role of STIM proteins is their participation in store-operated Ca^2+^ entry (SOCE) through SOC channels that are embedded in the plasma membrane (PM). Upon the stimulation of cells, after Ca^2+^ is released from the ER, STIM proteins are activated and transported to junctions between the PM and ER [12, 13], which in neurons extend from the cell body to presynaptic terminals, axons, dendrites, and dendritic spines [14]. In the junctions, STIM proteins bind and activate calcium release-activated calcium channel protein 1 and 2 (Orai1 and Orai2) proteins, forming complexes [3, 15–18]. In the rat cortex, SOCE is mainly triggered by Orai1–STIM1 complexes, and in the mouse brain by Orai2 and STIM2 (cortex and hippocampus; [6, 19]) or by Orai2 and STIM1 (cerebellum; [20]). In contrast, the dominant Orai homolog in astroglia is Orai3 but not Orai1 [21]. The resulting STIM–Orai heterocomplexes lead to the opening of highly Ca^2+^-selective SOC channels, allowing the influx of Ca^2+^ ions into the cytoplasm of neurons [3, 4, 8, 20, 22]. Although STIM1 is activated after complete emptying of the ER of Ca^2+^ ions, STIM2 is directed to the cell membrane after a slight decrease in Ca^2+^ level in the ER [4]. In addition to being located in the ER and ER–PM junctions, the presence of STIM1 in the PM of non-excitable cells [23–26] and neurons [27] has also been suggested, but this requires further study.

The roles of STIMs in neurons are still debated. However, STIM-mediated SOCE plays an essential role in both resting and firing neurons. In resting neurons, it constitutes the main route of Ca^2+^ entry [5, 28]. After glutamate release and PM depolarization, the number of STIM1 puncta increases significantly [29], and its function is twofold. It regulates glutamate release from presynaptic sites [30–33] and complements post-synaptic Ca^2+^ influx, which is mediated mainly by voltage-gated Ca^2+^ channels (VGCCs) and ionotropic receptors, such as α-amino-3-hydroxy-5-methyl-4-isoxazolepropionic acid receptors (AMPARs) and *N*-methyl-D-aspartate receptors (NMDARs; [28, 34–36]). Furthermore, STIM1 was previously shown to promote the internalization of Ca_V_1.2 and Ca_V_1.3 VGCCs, resulting in the total loss of function of these channels [8, 37, 38]. In turn, STIM2 regulates AMPAR trafficking by increasing levels of the GluA1 subunit of AMPARs on the cell surface by stimulating its exocytosis and inhibiting endocytosis [39, 40]. In addition, we previously found that STIMs in rat cortical neurons in vitro can interact with AMPAR and NMDAR subunits to affect the influx of Ca^2+^ ions into neurons [41, 42]. The silencing of STIM proteins increases Ca^2+^ influx through NMDARs [42] suggesting that STIM proteins help regulate NMDAR function. However, the link between STIMs and NMDAR transport to and from the PM has not yet been established.

NMDARs are highly permeable Ca^2+^ channels in the brain [43]. Functional NMDARs consist of a combination of two obligatory GluN1 subunits and two GluN2 or GluN3 subunits [44]. GluN1 binds glycine/serine, and GluN2 binds glutamate/NMDA, which is necessary for receptor activation [45]. In the cortex and hippocampus, GluN2A and GluN2B are the main NMDAR subunits.

NMDAR proteins are synthesized in the ER and then directed to the Golgi apparatus [46]. As mature receptors, they translocate along dendrites to excitatory synapses, where they alternate between exocytosis to the synaptic membrane and endocytosis into early endosomes. Large NMDAR populations are also present in the cytoplasm in cell bodies, within the ER/Golgi of the soma and larger dendritic branches of neurons and in glial cells [36, 47]. Dynamic modulation of the number of post-synaptic NMDARs, which is strictly regulated by endocytosis and exocytosis, is believed to be the main mechanism of alterations of excitatory synaptic activity and plastic changes in the central nervous system [48]. The excessive stimulation of these receptors leads to excitotoxicity and may contribute to the development of acute neurodegenerative diseases (e.g., stroke, traumatic brain injury, and epilepsy) and chronic neurodegenerative diseases (e.g., Alzheimer’s disease, Parkinson’s disease, and Huntington’s disease) [49]. Conversely, excessive NMDAR activation also leads to its endocytosis, which reduces uncontrolled Ca^2+^ overload and could be neuroprotective [50]. However, the molecular mechanisms and proteins that are involved in this process are poorly understood. Learning about new proteins that may regulate this process is paramount to understanding it more thoroughly. Therefore, the present study investigated the involvement of STIMs in the process of NMDAR endocytosis in dendritic spines of rat cortical neurons using an integrative experimental approach.

## Materials and methods

### Primary cell culture

As we previously described [4], cortical neurons were prepared from 19-day-old Wistar rat embryos. Pregnant female Wistar rats were obtained from the Animal Care Facility of the Mossakowski Medical Research Institute of the Polish Academy of Sciences (Warsaw, Poland). Animal care was in accordance with the Directive of the European Communities Council (86/609/EEC). Briefly, embryonic rat cortices were dissected, collected, rinsed in ice-cold Hanks’ Balanced Salt Solution (Sigma) that was supplemented with 11 mM HEPES buffer solution (Gibco) and 100 U/ml/100 µg/ml penicillin/streptomycin solution (Gibco), and treated with trypsin (Gibco) for 37 min at 37 °C. The tissue was then rinsed and dissociated by pipetting. For the co-immunoprecipitation (Co-IP) and biotinylation assays, neurons were seeded in 100 mm Biocoat poly-D-lysine (PDL) Cellware dishes (Corning) at a density of 10 × 10^6^ cells/dish. For isolation of the synaptosomal fraction and the preparation of cell lysates, neurons were seeded on PDL-precoated 6-well plates (Corning) at a density of 1.2 × 10^6^ cells/well. For the enzyme-linked immunosorbent assay (ELISA), neurons were seeded at a density of 3 × 10^5^ on 24-well plates that were precoated with PDL (Corning). For immunofluorescence and whole-cell patch clamp, neurons were seeded at a density of 2.2 × 10^5^ per 13 mm glass coverslip that was coated with a mixture of laminin (1.25 µg/ml; Roche, Mannheim, Germany) and PDL (37.5 µg/ml; Sigma-Aldrich, St. Louis, MO, USA) in 24-well plates. Neurons were grown in Neurobasal medium (Gibco, Paisley, UK) that was supplemented with 2% B27 (Gibco), 0.5 mM glutamine (Sigma), 12.5 µM glutamate (Sigma), and penicillin/streptomycin solution (Gibco) at 37 °C in a humidified atmosphere with 5% CO_2_/95% air. On day in vitro 3 (DIV3), half of the medium was replaced with a glutamate-free growth medium that contained the non-neuronal cell proliferation inhibitor CultureOne Supplement (Gibco). The experiments were performed after DIV10.

### Lentiviral production and neuronal transduction

HEK 293 T/17 cells (American Type Culture Collection) were grown in Dulbecco’s Modified Eagle Medium (DMEM) that was supplemented with 10% fetal bovine serum (FBS) and penicillin/streptomycin solution (Gibco) at 37 °C in a humid environment with 5% CO_2_/95% air. The viruses were prepared by the calcium phosphate transfection method. Three days after transfection, supernatants were collected, filtered through 0.45 μm membranes, concentrated in Vivaspin 100 kDa units (Sartorius) in a swing-out rotor at 3000 × *g*, aliquoted, and stored at −80 °C until needed.

Four commercially available Rat 29-mer target-specific short-hairpin RNA (shRNA) constructs (shStim1 C, shStim1 D, shStim2 C, and shStim2 D) in pLenti-green fluorescent protein vectors (GFP; Origene, Rockville, MD, USA) were used to knockdown STIM proteins. The targeting sequences for STIMs were as follows: GGATAATGGCTCTATTGGTGAGGAGACAG (TL707032C, shStim1 C), CTTCCAATGGTAGCCATCGGCTGATTGAG (TL707032D, shStim1 D), TTAGCCAGAAGCAGTAGTTTATGCCGCTC (TL704348C, shStim2 C), AGTCTGGAAGCACTTCAGACAATACATAA (TL704348D, shStim2 D). Scramble shRNA cassette in pLenti-GFP (scrRNA; Origene) was used as a control. The non-effective control sequence was 5' GCACTACCAGAGCTAACTCAGATAGTACT 3'. Neurons were transduced on DIV5 to knockdown STIM, and the experiments were performed 5 days after transduction (DIV10).

### Experimental conditions

Short-term neuronal overactivation was induced by 50 µM NMDA (Alomone) and 100 µM glycine (Sigma) for 15 min at 37 °C [51] in modified extracellular solution that was composed of 10 mM HEPES, 1.3 mM CaCl_2_, 110 mM Na_2_SO_4_, 5 mM Cs_2_SO_4_, 0.75 mM Na_2_HPO_4_, and 10 mM glucose (pH 7.4); [42]. To prevent cell damage during NMDAR overactivation, K^+^ and Cl^−^ were replaced by Cs^+^ and SO_4_^2−^, respectively [52].

Cell viability was estimated by measuring lactate dehydrogenase that was released to the extracellular solution using the CytoTox-ONE Homogenous Membrane Integrity Assay (Promega, Madison, WI, USA). Lactate dehydrogenase that was released by shStim- or scrRNA-transduced neurons was normalized to wild-type cells. Lactate dehydrogenase that was released by NMDA- and glycine-treated neurons was normalized to control cells.

### Total protein isolation

To prepare cell lysates, scrRNA- or shStim-transduced neurons were centrifuged at 1500 × *g* for 5 min at 4 °C. The pellet was lysed in buffer (pH 7.5), that contained 50 mM Tris, 150 mM NaCl, 0.1% SDS, 0.5% sodium deoxycholate (DOC), 1% NP-40, 1 mM phenylmethylsulfonyl fluoride, and complete mini EDTA-free Protease Inhibitor Cocktail (Roche), for 30 min on ice and centrifuged at 17,000 × *g* for 15 min at 4 °C. The supernatant was collected, and total protein concentration was measured using Bradford reagent (Sigma). Laemmli buffer was added, and the samples were boiled and resolved by SDS-PAGE.

### Western blot

Equal amounts of proteins were analyzed by 10% sodium dodecyl sulfate-polyacrylamide gel electrophoresis (SDS-PAGE), transferred to a nitrocellulose membrane (Amersham), and stained for total protein (Ponceau S staining). Membranes were blocked with 5% nonfat dried milk in TBS with 0.1% Tween 20 (TBST) and then incubated with the following primary antibodies: mouse anti-GluN1 (Thermo Fisher, Catalog No. 32-0500, 1:200), rabbit anti-GluN2A (Alomone, Catalog No. AGC-002, 1:200), rabbit anti-GluN2B (Proteintech, Catalog No. 21920-1-AP, 1:200), rabbit anti-STIM1 (Proteintech, Catalog No. 11565-1-AP, 1:200), rabbit anti-STIM2 (Proteintech, Catalog No. 21192-1-AP, 1:200), mouse anti-EEA1 (BD, Catalog No. GT10811, 1:200), rabbit anti-post-synaptic density 95 (PSD95; Alomone, Catalog No. APZ-009; Cell Signaling, Catalog No. 2507S, 1:200), rabbit anti-N-cadherin (Proteintech, Catalog No. 22018-1-AP, 1:2000), rabbit anti-α-tubulin (Proteintech, Catalog No. 11224-1-AP, 1: 200 000), rabbit anti-Na^+^/K^+^-ATPase (Proteintech, Catalog No. 14418-1-AP, 1:1000), rabbit anti-calnexin (Proteintech, Catalog No. 10427-2-AP, 1:1000), and rabbit anti-LDH (Proteintech, Catalog No. 14824-1-AP, 1:1000). The specificity of ProteinTech anti-STIM1 and anti-STIM2 antibodies has been verified earlier several times in [53, 54] with the use of STIM1 siRNA or in [55, 56] with the use of STIM2 siRNA. Specific band for STIM1 and STIM2 was detected at approximately 90 kDa and 100 kDa, respectively, as observed by other authors using the same antibody from ProteinTech [53, 57–59]. The blot confirming the specificity of the antibody using shRNA presented in Fig. S1A, shows that shStim1 does not reduce STIM2 and shStim2 does not reduce STIM1. Membranes were then incubated with anti-mouse (Sigma-Aldrich, 1:4000) or anti-rabbit (Sigma-Aldrich, 1:5000) horseradish peroxidase-conjugated secondary antibodies. Bound antibodies were visualized by the chemiluminescence detection reagent Amersham ECL (Cytiva). If necessary, the membranes were stripped and reprobed. The samples were imaged using the Fusion FX imaging system (Vilber Lourmat, Marne-la-Vallée, France) and quantified using Image Studio Lite 5.2 software. The intensity of bands was normalized to total protein densities that were acquired by Ponceau S staining, corresponding to the same lane [60, 61], and quantified using ImageJ software with the Gel analyzer feature (National Institutes of Health, Bethesda, MD, USA) or to the intensity of α-tubulin (cell lysate) or N-cadherin (surface proteins) bands.

### Synaptic protein isolation

Synaptosomal fractions from wild-type neurons or scrRNA- or shStim-transduced neurons were isolated using Syn-PER Synaptic Protein Extraction Reagent (Thermo Scientific, Catalog No. 87793). Neurons were centrifugated at 1200 × *g* for 10 min at 4 °C to dispose of cellular debris. The supernatant was then centrifuged at 15,000 × *g* for 20 min at 4 °C, and the pellet was suspended in Syn-PER reagent. This procedure yielded synaptosomes that contained pre- and post-synaptic proteins.

### Co-immunoprecipitation

For the Co-IP of proteins, neurons were collected and lysed for 1.5 h. Lysates were centrifuged at 15,000 × *g* for 15 min at 4 °C, and the supernatant was collected and mixed with Protein G-Agarose beads (Roche). Precleared lysates (700 µg) were incubated overnight with protein G-Agarose that was preincubated for 3 h with the following antibodies: rabbit anti-GluN2A (Alomone, Catalog No. AGC-002) or rabbit anti-GluN2B (Proteintech, Catalog No. 21920-1-AP). Rabbit anti-immunoglobulin G (IgG) antibody (Merck, Catalog No. 12-370 and 12-371) was used as a negative control. The precipitated proteins were eluted and separated by SDS-PAGE.

### Cell surface protein quantification assays

In wild-type neurons, surface proteins were isolated using the cell-surface protein isolation kit (Abcam, Catalog No. ab206998). Briefly, surface proteins were biotinylated with a solution of Sulfo-NHS-SS-Biotin for 30 min at 4 °C. Thereafter, a quenching solution was added to bond excess biotin. Cells were centrifuged, and the pellet was washed with Tris-buffered saline (TBS) and lysed. On average, we yielded 400 µg of proteins during one experiment in 400 µl of the lysis buffer. According to supplier protocol, 50 µl was left as „lysate” and 350 µl was incubated with 150 µl of streptavidin beads for 2 h with end-over-end mixing at 4 °C. In subsequent steps of the kit protocol, it is not possible to determine the protein concentration due to the buffer formulation nor to estimate what percentage of protein was collected by the beads. The surface proteins were eluted from the beads using 60 µl phosphate-buffered saline (PBS) with the strong reducing agent 0.1 M dithiothreitol to break the Sulfo-NHS-SS-biotin disulfide bonds, releasing the isolated proteins into solution and subjecting them to initial degradation and solubility. Laemmli buffer was then added in the presence of SDS for complete denaturation. Two gels were loaded with 25 µl of both the "lysate" and "surface protein" fractions. Approximately, the amount of protein in the eluted surface fraction is 2.2 times lower than in the lysates, as shown by Ponceau staining of the membrane (Fig. S2A).

Due to material limitations, in experiments on primary neurons in culture that were transduced with scrRNA or shStim (1*n* = cerebral cortex from 30 rat embryos), surface proteins were analyzed by a quantitative cell-based ELISA [62]. Neurons were fixed in 4% paraformaldehyde and 4% sucrose in PBS. The permeabilization step was omitted, because only the NMDAR surface was measured. Nonspecific binding was then blocked by 5% bovine serum albumin in PBS. Subsequently, cells were incubated with primary antibodies for the extracellular epitope of GluN1 (guinea pig, Alomone, Catalog No. AGP-046, 1:100), GluN2A (mouse, Abcam, Catalog No. ab240884, 1:100), or GluN2B (rabbit, Alomone, Catalog No. AGC-003, 1:100) in blocking solution for 2 h. Cells were then incubated with horseradish peroxidase-conjugated anti-guinea pig, anti-mouse, or anti-rabbit secondary antibody (Sigma-Aldrich, 1:2000) for 1 h. Afterward, 1-Step Turbo TMB-ELISA Substrate Solution (Thermo Fisher, Catalog No. 34022) was added, incubated for 30 min in the dark, and terminated by 1 M H_2_SO_4_. Absorption was measured at 450 nm using a Tecan INFINITE M1000 PRO plate reader with dedicated software (Tecan Group, Mannedorf, Switzerland). For normalization, the staining of cell nuclei with Hoechst 33,342 (Thermo Fisher, 1:10,000, 5 min) was used, the fluorescence of which was measured at an excitation wavelength of 358 nm and emission wavelength of 461 nm.

### Immunofluorescence and image acquisition

For immunofluorescent staining, neurons were fixed in ice-cold 4% paraformaldehyde and 4% sucrose, permeabilized with 0.05% saponin (Sigma) in PBS, and blocked with 2% normal goat serum in PBS. The cells were then incubated at room temperature for 2 h with the following antibodies: rabbit anti-GluN1 (Abcam, Catalog No. ab17345, 1:50), mouse IgG2a anti-GluN1 (Thermo Fisher, Catalog No. 32-0500, 1:50), rabbit anti-GluN2A (Alomone, Catalog No. AGC-002, 1:50), mouse IgG2a anti-GluN2A (Abcam, Catalog No. ab240884, 1:50), rabbit anti-GluN2B (Alomone, Catalog No. AGC-003, 1:50), mouse IgG anti-GluN2B (Abcam, Catalog No. ab93610, 1:50), rabbit anti-STIM1 (Proteintech, Catalog No. 11656-1-AP, 1:50), rabbit anti-STIM2 (Alomone, Catalog No. ACC-064, 1:50), mouse IgG anti-EEA1 (BD, Catalog No. GT10811, 1:50), and chicken anti-MAP2 (Thermo Fisher, Catalog No. PA1-16,751, 1:500) diluted in blocking solution with 0.05% saponin. Immunoreactivity was detected with anti-mouse IgG Alexa Fluor 488-, anti-mouse IgG2a Alexa Fluor 488-, anti-chicken Alexa Fluor 568-, or anti-rabbit Alexa Fluor 647-conjugated secondary antibodies (Thermo Fisher). Nuclei were stained with Hoechst 33,342. Coverslips were mounted on slides with ProLong Gold Antifade Mountant (Thermo Fisher). For the detection of surface proteins, the permeabilization step was omitted, and incubation with antibodies without detergent was performed.

To label surface NMDAR subunits in STIM1/STIM2-silenced or control (scrRNA) neurons [63], cells were fixed and incubated for 2 h at room temperature with rabbit antibody directed against the N-terminal extracellular epitope of GluN1 (Alomone, Catalog No. AGC-001, 1:50), GluN2A (Alomone, Catalog No. AGC-002, 1:50), or GluN2B (Alomone, Catalog No. AGC-003, 1:50). After washing, neurons were incubated with anti-rabbit IgG Alexa Fluor 647 secondary antibody (Thermo Fisher, 1:500) at room temperature for 45 min. Neurons were then permeabilized, blocked, and incubated with the same primary rabbit antibody against GluN1, GluN2A, or GluN2B and mouse antibody against EEA1 (BD, Catalog No. GT10811, 1:50). Immunoreactivity was visualized using a combination of anti-rabbit and anti-mouse secondary antibodies conjugated to Alexa Fluor 405 (Thermo Fisher) and Alexa Fluor 568 (Thermo Fisher), respectively.

Immunostained cells were viewed under a Zeiss LSM780 Axio Observer confocal microscope (Carl Zeiss AG, Oberkochen, Germany). Images were acquired using a 63 × Alpha Plan-Apochromat oil immersion objective. Image resolution was 1024 × 1024 pixels. The laser power and detector gain were adjusted to obtain the maximum signal without oversaturation and cross-fluorescence with minimum background signal. Images were processed with ZEN 3.0 (blue edition) and deconvoluted using the ImageJ software (National Institutes of Health, Bethesda, MD, USA). Manders’ co-localization coefficient (M1) was used to estimate the level of protein co-localization [64].

### Whole-cell patch-clamp recording

NMDAR currents were recorded in the whole-cell configuration of the patch-clamp technique at room temperature. Series resistance was 3–13 MΩ (constant during recording, controlled before and after recording). During recordings, neurons were voltage-clamped at −60 mV and submerged into the solution stream that was supplied by a manually controlled multi-barreled perfusion system (~ 1 ml/min). The intracellular solution comprised 140 mM CsF, 5 mM BAPTA, 1 mM CaCl_2_, 4 mM MgCl_2_, 10 mM HEPES, and 2 mM Na_2_ATP (pH 7.3). The extracellular solution contained 140 mM NaCl, 1.3 mM CaCl_2_, 5 mM KCl, 25 mM HEPES, 33 mM glucose, and 500 nM tetrodotoxin (pH 7.35; [51]). To prevent desensitization of the NMDA channels, the currents were evoked by 4 subsequent 4-s applications of 50 µM NMDA and 100 µM glycine, separated by 5-min intervals (total experiment time 15 min). Each of these four records is recorded from one cell. Shown are recordings of currents in one cell obtained after subsequent doses of NMDA and glycine. To block NMDAR endocytosis, the glycine site antagonist MDL29951 (5 µM) was used. The compounds were dissolved in extracellular solution and applied to the recording chamber via a perfusion system. NMDAR currents were recorded with an Axopatch 1-D amplifier. Data were digitized using DigiData1200A, filtered (2 kHz), and acquired using pClamp10.6 software.

### Statistical analysis

The statistical analysis was performed using Prism 10 software (GraphPad, San Diego, CA, USA). The results are expressed as the mean ± standard error of the mean (SEM) of three-to-six independent experiments. Statistical significance was determined by unpaired t test, or by one- or two-way Anova + Tukey's post hoc test for multiple comparisons, as indicated in figure legend. Values of *p* < 0.05 were considered statistically significant.

## Results

### NMDAR endocytosis occurs shortly after its overactivation

**Fig. 1:**
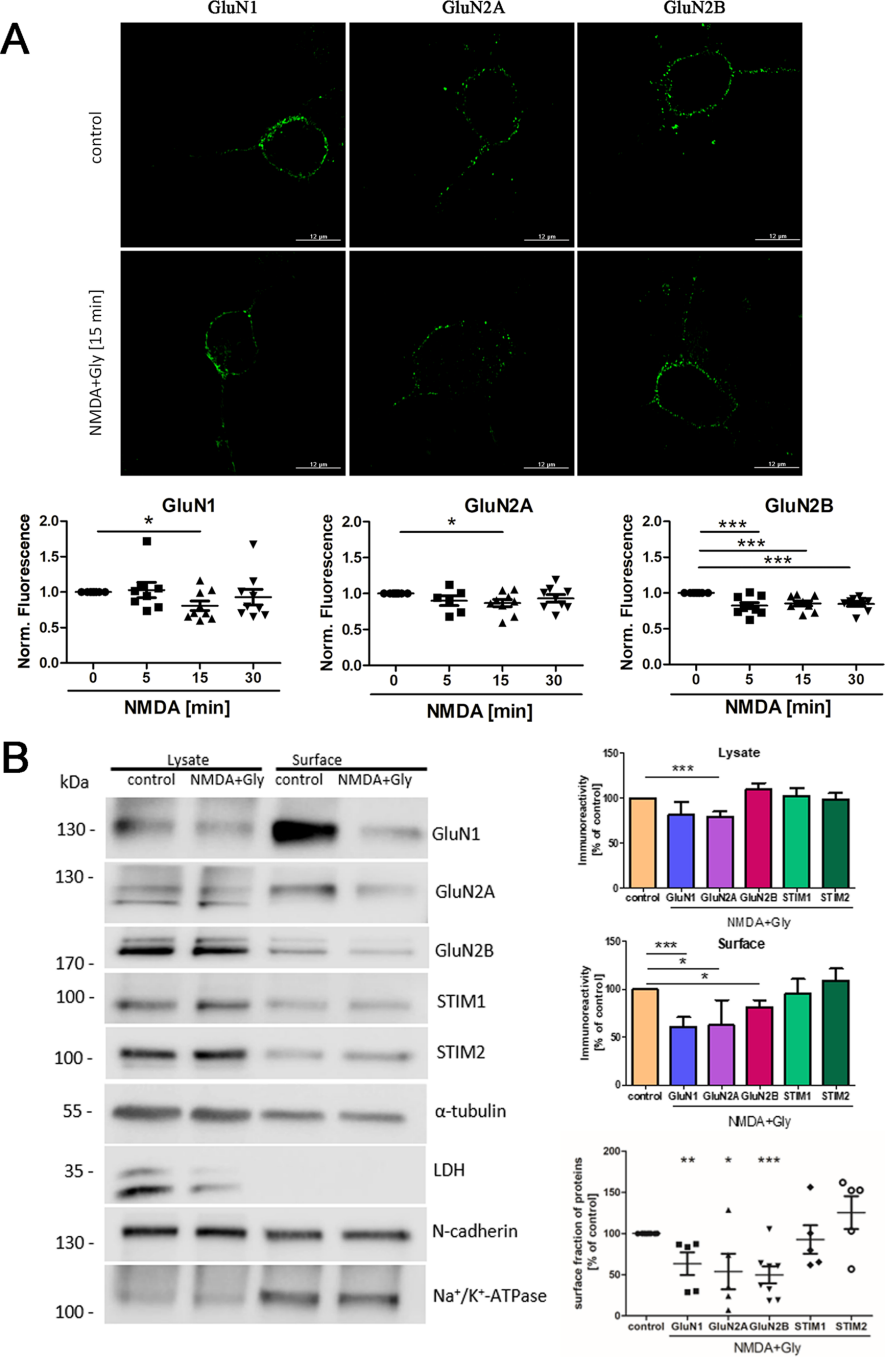
Short-term NMDAR overactivation induces its internalization from the plasma membrane. **A** Immunofluorescence of surface NMDAR subunits (GluN1, GluN2A, and GluN2B) in neurons in vitro that were treated with 50 μM NMDA and 100 μM glycine (NMDA) for 15 min and stained with antibodies without permeabilization. All deconvoluted confocal microscopy images were taken from a single optical section in the middle of the cell. Scale bar = 12 μm. Graphs represent quantification of the effect of 5, 15, and 30 min of treatment on the surface localization of NMDAR subunits. Data are expressed as the mean ± SEM of three independent experiments relative to fluorescence intensity values obtained in untreated control cells, which were arbitrarily assigned a value of 1. **B** Representative Western blots and densitometric analysis of NMDAR subunits and STIM proteins in the total cell lysate and on the cell surface in control neurons and neurons treated with 50 µM NMDA and 100 µM glycine (NMDA + Gly) for 15 min in vitro. The Western blot signals of α-tubulin and N-cadherin were used as loading controls for the lysate and surface proteins, respectively, to which the bar graphs were normalized. LDH and Na^+^/K^+^-ATPase were used as cytosolic and PM markers, respectively. The bottom dot plot shows the quantification of biotinylated proteins as a fraction of the total content of a given protein measured in the cell lysate as calculated in [104]. For each protein, the fraction biotinylated after NMDA and glycine treatment is shown as a percentage of its presence on the surface of control (untreated) cells from the same experiment. The data are expressed as the mean ± SEM of four-to-eight independent experiments. *p < 0.05, **p < 0.01, ***p < 0.001 (unpaired *t* test)

Although prolonged NMDAR overactivation has been thoroughly studied in recent years [63, 65–67], the immediate effects of acute NMDAR overactivation have only been described selectively [51, 52, 67]. Therefore, we tested whether short-term NMDAR overactivation induces NMDAR internalization. As shown in Fig. 1A, in rat cortical neurons that were treated for 5, 15, or 30 min with 50 µM NMDA and 100 µM glycine, the intensity of immunofluorescence of the extracellular GluN1, GluN2A, and GluN2B subunits was significantly weaker than in control neurons. The greatest difference occurred 15 min after treatment. To rule out the possibility that the above conditions lead to neuronal cell death, a neuronal viability assay was performed, which showed no difference between the tested groups (Fig. S3).

To corroborate the reduction of surface NMDARs after 15 min of NMDAR overactivation, external membrane proteins were biotinylated with the use of a commercial cell surface protein isolation kit, and levels of both surface and total proteins were analyzed by Western blot (Fig. 1B). NMDA and glycine treatment significantly reduced surface GluN1 and GluN2B levels to 61.02% ± 9.86% and 81.61% ± 7.22%, respectively, whereas corresponding total protein levels of GluN1 and GluN2B remained unchanged (Fig. 1B). These results suggest that short-term NMDAR overactivation increases the internalization of GluN2B-containing NMDARs without affecting the total amount of these receptors. Although the total level of GluN2A was significantly reduced by 20.75% ± 5.62%, the amount of GluN2A on the surface was reduced by 37.31% ± 15.71%, suggesting that the predominant effect on GluN2A-containing NMDARs was its internalization and not degradation. The % of proteins that were pulled down by the streptavidin beads is shown in the third graph as a scatter plot, where the biotinylated proteins are divided by the total content of a given protein measured by densitometry in the cell lysate, and is consistent with the plot showing surface proteins.

Moreover, we also observe the presence of STIM1 (similar to [27]) and STIM2 proteins in the surface fraction, which may suggest their association with PM or their presence in PM in our experimental conditions. Other possibility is that STIM can be derived from protein complexes that are pulled together by biotinylated proteins (Fig. 1B). The results of subcellular fractionation show (Fig. S2B) that despite the approx. equal volume of calnexin, the level of STIM proteins is higher in PM than in cytosolic fraction (Cytosol), which may suggest their presence also in PM (not only in the cytosolic fraction containing ER). Also, the enrichment of the PM marker, N-cadherin, in the PM fraction compared to the total membrane fraction (TM) and the cytosolic fraction (CF) is evident (Fig. S2B). Additionally, both the total and surface amounts of STIM proteins remained unchanged after NMDA and glycine treatment.

A small amount of tubulin in the surface fraction is visible, which may indicate that biotinylated proteins can pull membrane-bound protein complexes (e.g., cytoskeletal proteins such as tubulin, which can penetrate deeply into the plasma membrane as an integral membrane protein [68]). Therefore, the blotting the intracellular protein lactate dehydrogenase B (LDH, cytosolic marker) ensure that the neurons remained intact during the labeling step and that a biotinylation reagent was unable to enter the cells and label the internal proteins. We observed no LDH signal in the plasma membrane fraction, which can prove the specificity of the biotinylation (Fig. 1B). No enrichment of the N-cadherins or NMDAR subunits is seen in the surface fraction in comparison to the total lysates, since only a small part of the proteins remained after the purification procedure. Similar results for a small enrichment of the surface fraction in cadherin using the same reagent kit have been showed elsewhere [69]. To confirm that the surface fraction is enriched in surface proteins, a blot with anti-Na^+^/K^+^-ATPase, which is considered as the most specific non-glutamate marker expressed on the surface of neurons, was performed. In this case, a 2.3-fold enrichment is evident (Fig. 1B).

**Fig. 2:**
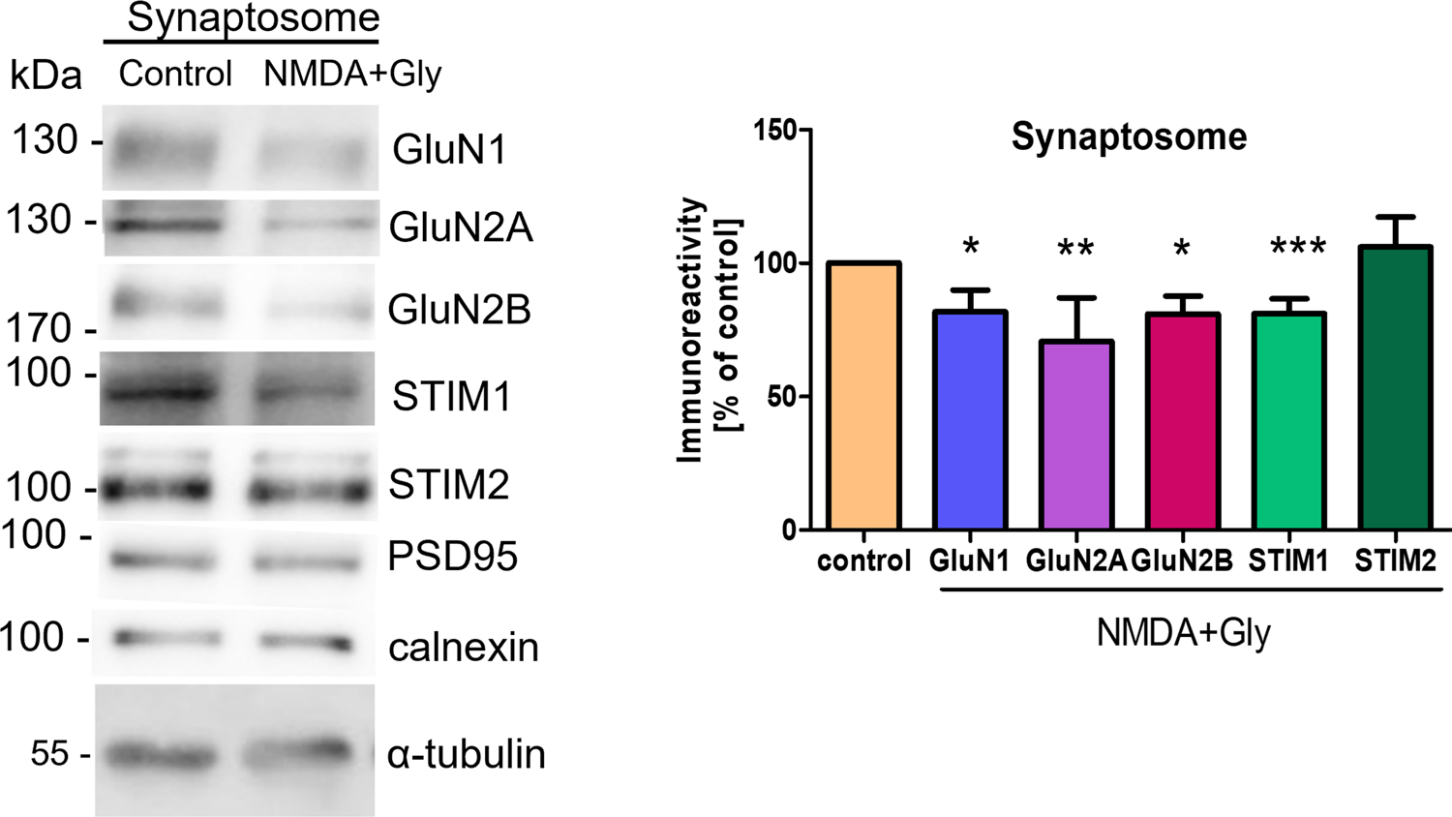
Short-term NMDAR overactivation induces its internalization from the synaptosome. Representative Western blot and densitometric analysis of NMDAR subunits (GluN1, GluN2A, and GluN2B) and STIM proteins in the synaptosomal fraction that was isolated from primary neurons in vitro. PSD95 and calnexin were used as synaptosome and ER markers, respectively. Total protein levels, determined by Ponceau S staining (see "Materials and methods" section), were used as loading controls. The data are expressed as the mean ± SEM of four-to-six independent experiments. *p < 0.05, **p < 0.01, ***p < 0.001 (unpaired *t*-test)

In addition to the NMDAR subunits, STIM proteins are also present in the control synaptosomal fraction (Fig. 2). These results correlate well with the previous results that STIM1 and particularly STIM2 proteins localize to dendritic spines and synapses and are present in synaptosomes [17, 32, 33, 39, 70–72], which we will discuss later. Notably, ER in the dendritic spines of the rat hippocampal neurons occupied 12–40% of the spine area [73]. In addition, a higher level of STIM2 was evident in the synaptosomal fraction (along with the ER marker calnexin) and only a minimal amount was present in the cytosolic fraction when both fractions were processed together and loaded onto gels in equal amounts (20 µg) (Fig S4). In this condition, the PSD95 enrichment in the synaptosomal fraction was 2.25-fold. This is another argument supporting the localization of STIM2 in synaptosomes.

Densitometric analysis revealed that short-term NMDA and glycine treatment significantly reduced synaptosomal GluN1, GluN2A, and GluN2B levels to 81.84% ± 8.06%, 70.64% ± 16.43%, and 80.96% ± 6.77%, respectively (Fig. 2). The results are consistent with the data in Fig. 1 and suggest that NMDARs are internalized from the PM and synaptosome. Although treatment with NMDA and glycine did not influence the amount of synaptosomal STIM2, it significantly decreased the amount of STIM1 in the synaptosome. Moreover, the amount of STIM1 in the PM remained unchanged (Fig. 1B), suggesting a migration of STIM1 from synaptic to extrasynaptic sites.

**Fig. 3:**
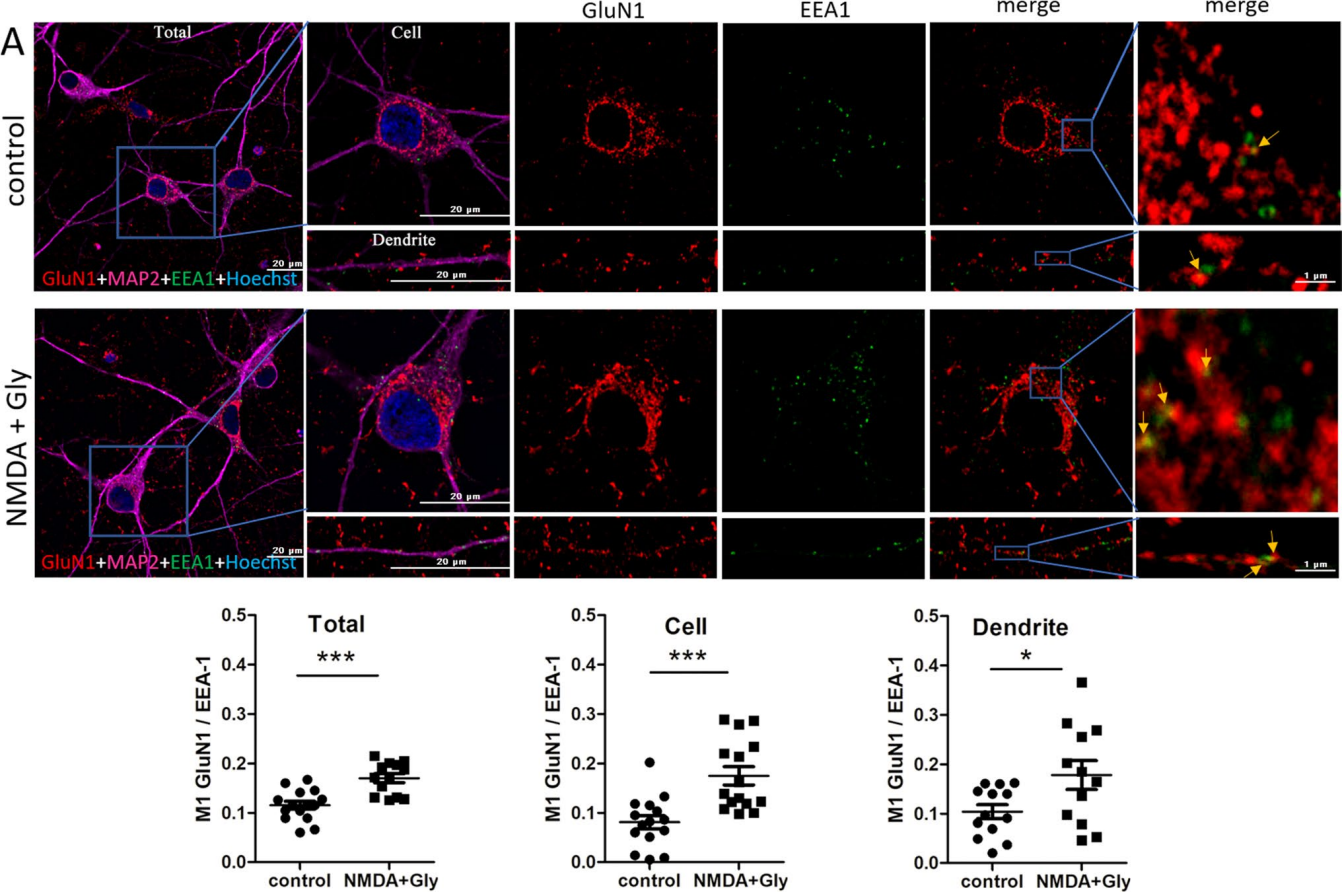

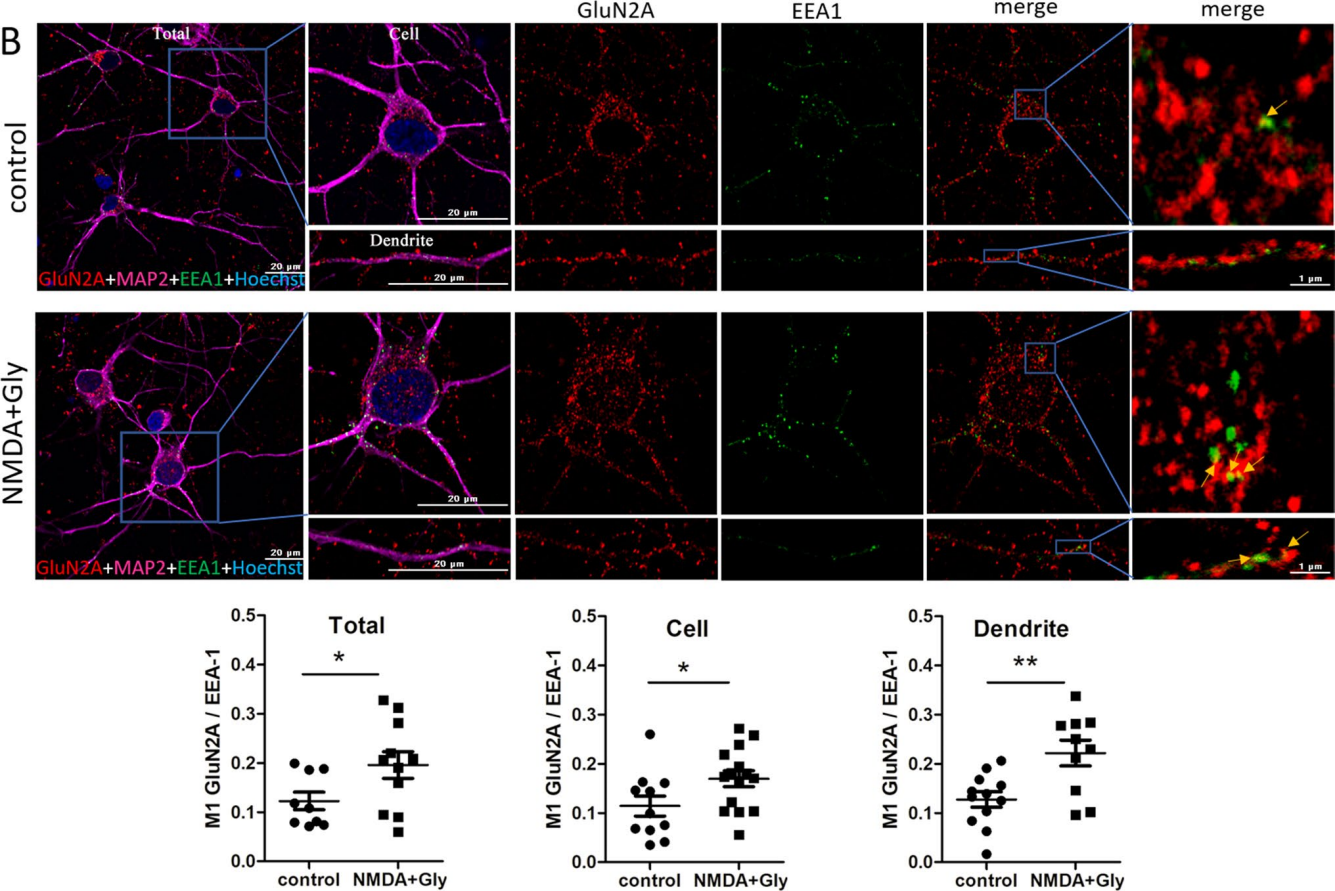

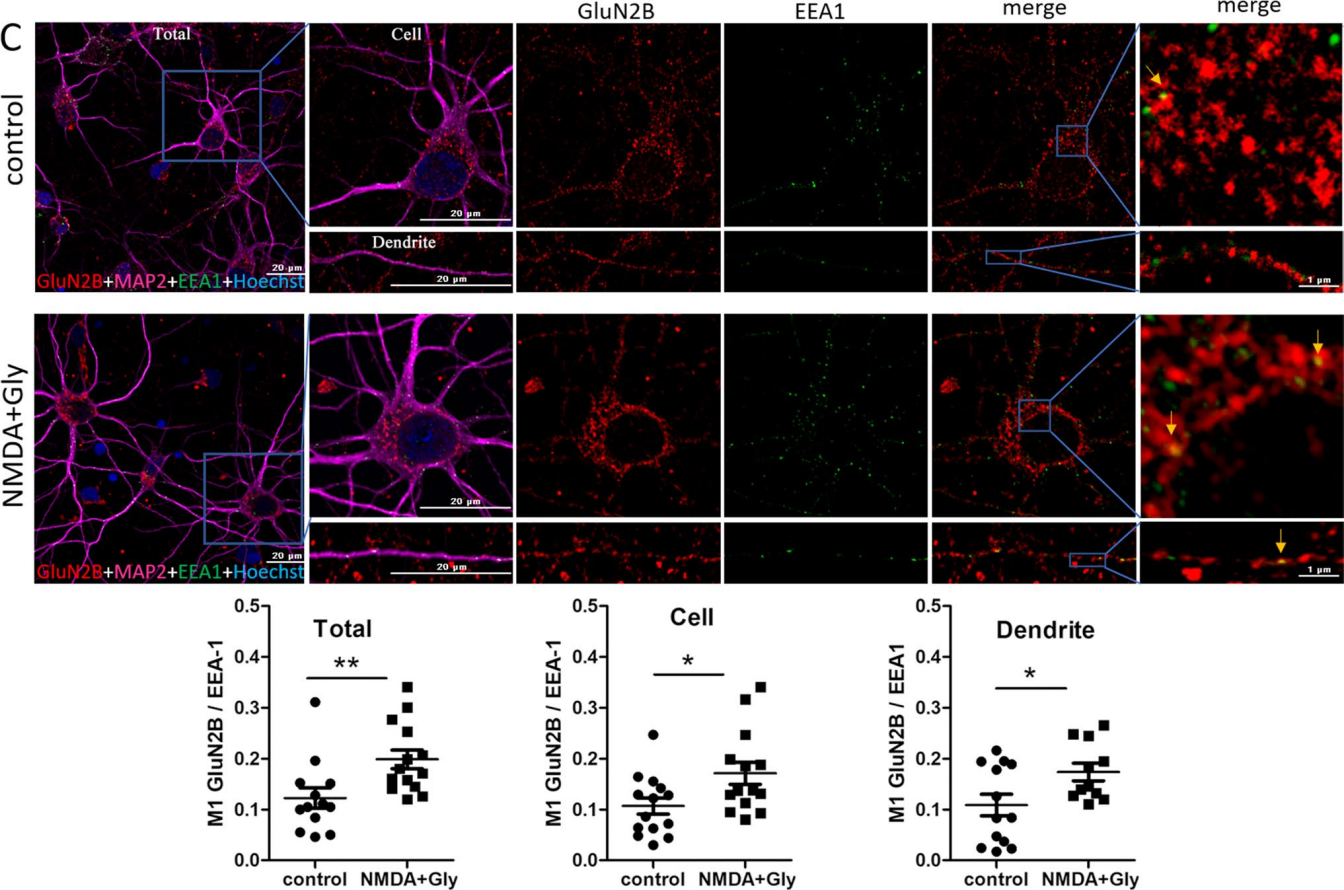
Short-term NMDAR overactivation increases the co-localization of GluN1, GluN2A, and GluN2B with the early endosome marker EEA1. **A**–**C** Representative deconvoluted confocal microscopy images of NMDAR subunits with early endosome marker EEA1 before and after the NMDA and glycine treatment of neurons in vitro and quantification of the co-localization points using Manders’ co-localization coefficient (M1). The co-immunostaining of GluN1, GluN2A, or GluN2B (red), EEA1 (green), and nuclei (blue) is shown. Staining with anti-MAP2 (neuron-specific cytoskeletal protein) antibody (magenta) was used to identify neurons and individual dendrites on which yellow co-localization points were counted. The first and last columns (higher magnification) show GluN1, GluN2A, and GluN2B labeling overlapped with EEA1 labeling. Arrows indicate representative EEA1-positive puncta that contain endocytosed NMDARs. All images were taken from a single optical section in the middle of the cell. Scale bar = 20 µm or 1 µm. M1 was calculated from the entire field of view (Total), single cell (Cell), and dendrites (Dendrite). The data are expressed as the mean ± SEM of three independent experiments. *p < 0.05, **p < 0.01, ***p < 0.001 (unpaired *t* test)

We assumed that short-term NMDAR overactivation may increase NMDAR endocytosis into early endosomes. Thus, GluN1, GluN2A, and GluN2B were immunostained along with a neuronal marker, anti-microtubule-associated protein 2 (MAP2), and the early endosome marker EEA1 in neurons after NMDA and glycine treatment, and the co-localization rate was compared using Manders’ co-localization coefficient (M1; Fig. 3). An M1 value for GluN (Total) of approximately 0.12 means that 12% of the red pixels (11.5% for GluN1, 12.3% for GluN2A and 12.3% for GluN2B) that occupy most of the cell co-localize with the green ones (EEA1, significantly fewer dots compared to GluN staining). However, after induction of endocytosis, the M1 values increase to 0.17, 0.196, and 0.20, indicating that already 17%, 19.6%, and 20% of red GluN1, GluN2A, and GluN2B, respectively, co-localize with green endosomes [64] and this is a statistically significant increase (by 47%, 59%, and 62%, respectively). The difference was visible across the entire field of view (Total), cell body (Cell), and dendrites (Dendrite). Therefore, NMDARs underwent endocytosis shortly after the overactivation of neurons.

**Fig. 4:**
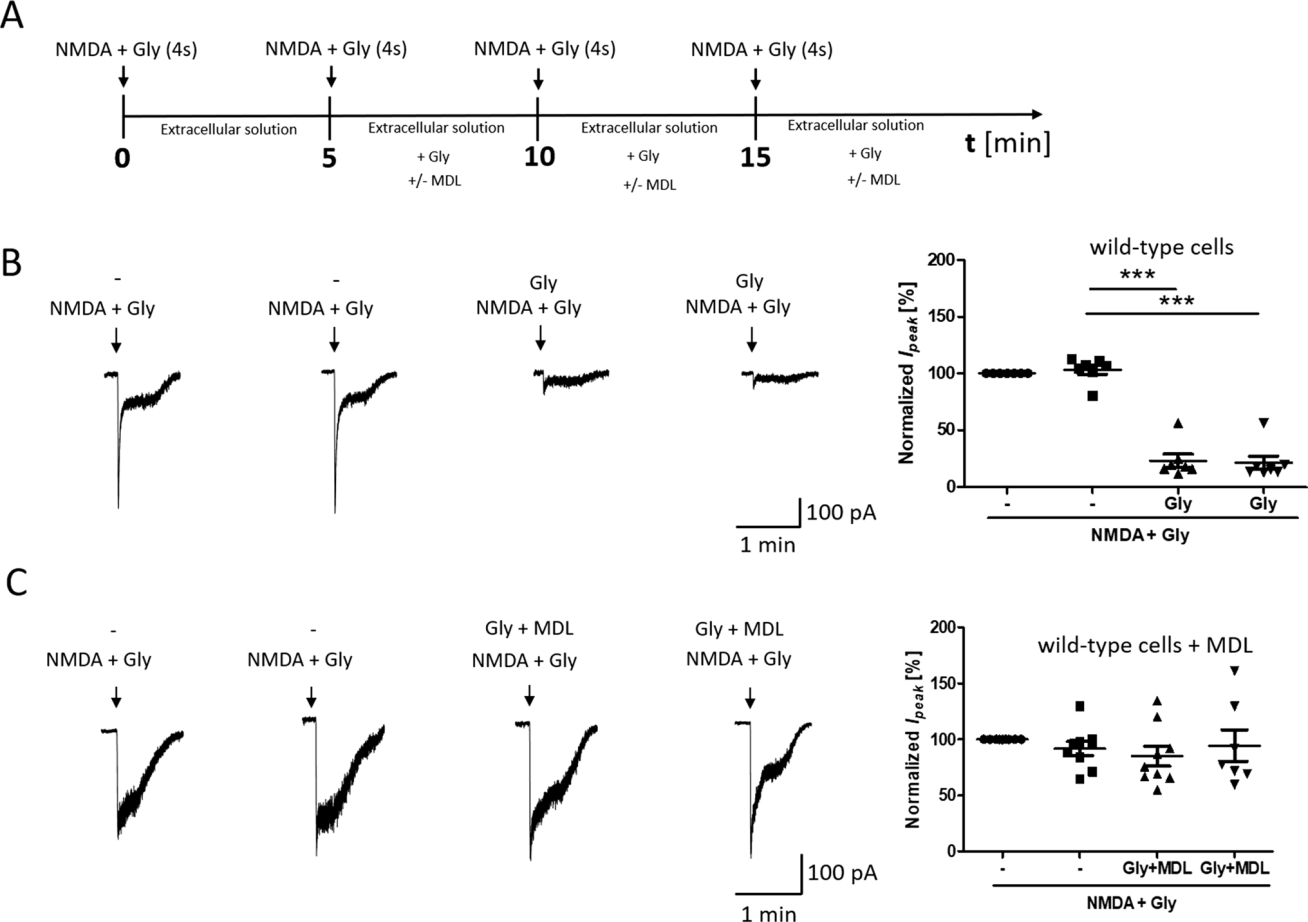
NMDAR overactivation-induced endocytosis decreases NMDAR-mediated ionic current. **A** Scheme of the experimental protocol. **B**, **C** Traces show examples of NMDAR currents that were recorded using protocols from one cell at a time. The plots show normalized NMDAR peak currents that were recorded in rat primary cortical neurons that were 4 times treated with 50 µM NMDA and 100 µM glycine (NMDA + Gly) for 4 s without (**B**) or with 5 µM MDL29951 (MDL) (**C**). Peak currents were normalized to the first response that was evoked by the applications of 50 µM NMDA and 100 µM glycine. The data are expressed as the mean ± SEM of three independent experiments. ***p < 0.001 (unpaired *t* test). *n* = 7–9 cells

To further confirm that the NMDAR is endocytosed shortly after its overactivation, another type of experiment was performed where whole-cell NMDAR currents were measured. Since the electrophysiology technique is sensitive to desensitization, while microscopy is not, the protocol was chosen, so that desensitization did not affect the measured effect attributed to internalization. Thus, to prevent NMDAR desensitization [74], 4 short 4-s activations of the receptor with 50 µM NMDA and 100 µM glycine (as in other experiments including Co-IP and immunofluorescence) with 5-min intervals [15 min in total] were used (Fig. 4A). As shown in Fig. 4B, repeated 4-s NMDA and glycine stimulation at 5-min interval did not change the amplitude of NMDAR currents (Fig. 4B, t5). However, the next activation of the NMDA receptor with 50 µM NMDA and 100 µM glycine after 5 min of incubation in buffer in the presence of glycine reduced the current amplitude to 23.00% ± 5.71% of the basal magnitude (Fig. 4B, t10), which is also observed after activation the NMDAR after another 5 min (Fig. 4B, t15).

Treatment with the NMDAR glycine site blocker MDL29951 did not result in the inhibition of NMDAR currents (91.86% ± 6.20%; Fig. 4C), suggesting that the NMDAR is internalized immediately after its overactivation. The variability of steady current recordings seen in Fig. 4B and C is most likely due to differences in perfusion rates and cell localization, i.e., whether the cell was located further or closer to the place where solution was administered to the microscope camera. In order for the steady-state current not to affect the analyzed results, the data in Fig. 4 were referred to the value of the peak current. The experiments were characterized by low variability of maximum currents in all control experiments.

To sum up, as the glycine site activation is essential for the initiation of endocytosis [51], our results suggest that internalization occurred through the mechanism of endocytosis.

### NMDAR endocytosis increases interactions between STIM2 and GluN2A and between STIM2 and GluN2B

**Fig. 5:**
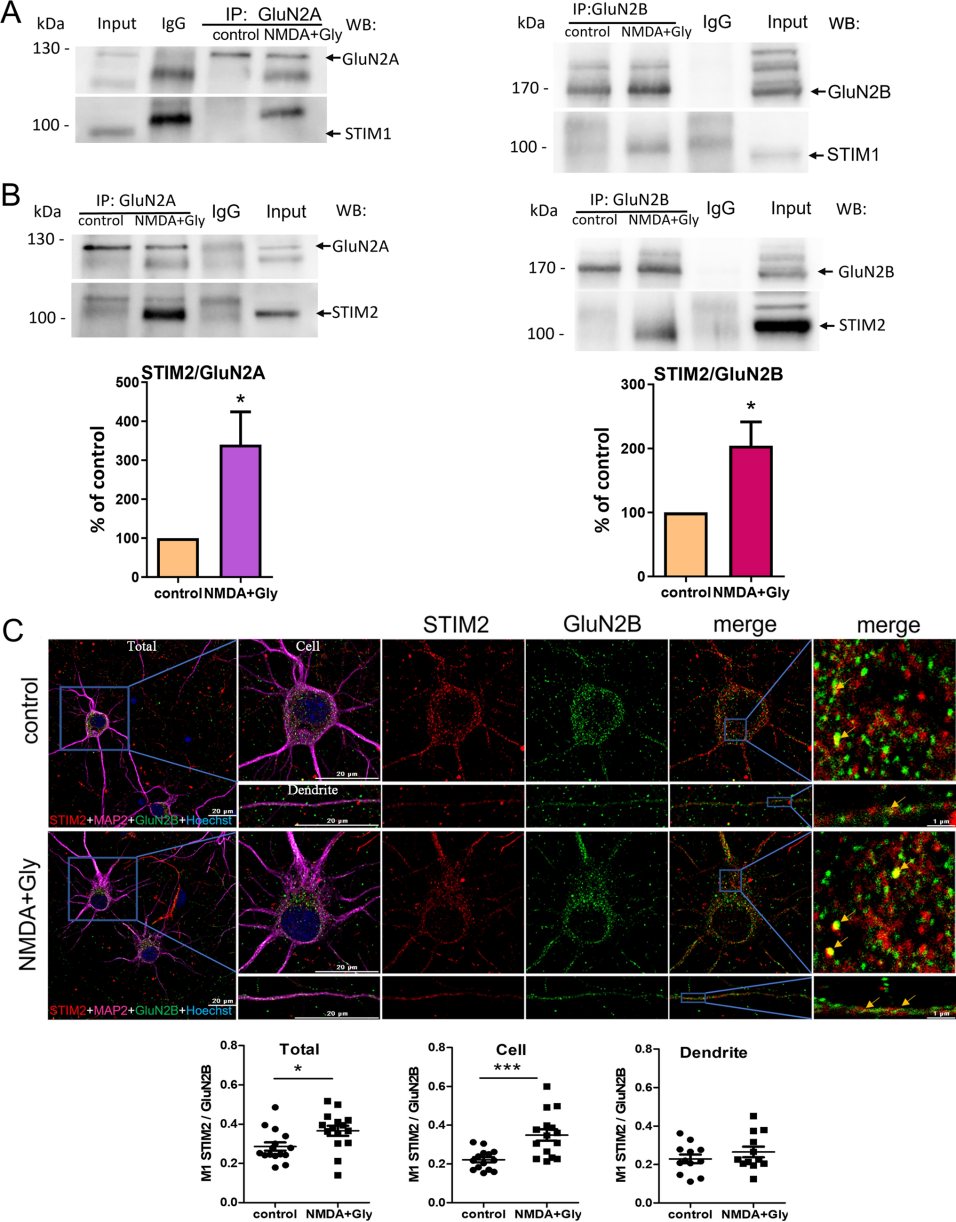
Short-term NMDAR overactivation increases interactions between STIM2 (but not STIM1) and GluN2A or GluN2B in cortical neurons in vitro. **A**, **B** Representative Western blots from Co-IP experiments and **B** quantification of interactions between STIM proteins and NMDAR subunits before and after the NMDA and glycine treatment of neurons. Immunoprecipitation was performed with anti-GluN2B, anti-GluN2A, and anti-IgG antibodies. The input represents the cell lysate. The fractions were immunoblotted with the indicated antibodies. The Co-IP bands were normalized to the level of the loading control (i.e., bands obtained after Western blot with the antibody that was used for immunoprecipitation). **C** Representative deconvoluted confocal microscopy images of STIM2 with GluN2B before and after the NMDA and glycine treatment of neurons. The co-immunostaining of STIM2 (red), GluN2B (green), MAP2 (magenta), and nuclei (blue) is shown. The first “merge” column shows STIM2 labeling overlapped with GluN2B labeling, and the last column shows its higher magnification. All images were taken from a single optical section in the middle of the cell. Scale bar = 20 µm and 1 µm. Quantification of co-localization points was performed using Manders’ co-localization coefficient (M1). M1 was calculated from the entire field of view (Total), single cell (Cell), and dendrites (Dendrite). The data are expressed as the mean ± SEM of three-to-four independent experiments. *p < 0.05, ***p < 0.001 (unpaired *t* test)

Since endogenous STIM proteins interact with NMDAR subunits [42], we sought to determine the influence of NMDAR overactivation on these interactions. As shown in Fig. 5B, there was a weak interaction between GluN2A and STIM2, and GluN2B and STIM2 in control neurons. These interactions changed after NMDAR endocytosis. Short-term NMDAR overactivation strongly increased the formation of complexes between STIM2 and GluN2A, and STIM2 and GluN2B to 339.9% ± 84.14% and 204.2% ± 37.22%, respectively (Fig. 5B). In contrast, association between STIM1 and GluN2A/GluN2B was not noted in our experimental conditions as visible on WB in Fig. 5A.

These relationships were further tested by immunostaining and Manders’ co-localization coefficient (M1). The most apparent difference in M1 was observed for GluN2B and STIM2 (Fig. 5C). Consistent with the Co-IP results, NMDAR endocytosis increased STIM2 co-localization with GluN2B and a significant difference was observed in the entire field of view (Total) and cell body (Cell) but not in dendrites (Dendrite). Additionally, the labeling of GluN2B in treated neurons appeared to be more punctate compared with the control. For STIM2-GluN2A, no changes in co-localization were observed after NMDA and glycine treatment (Fig. S5). Thus, the Co-IP and immunofluorescence data indicated that NMDAR overactivation and subsequent endocytosis primarily affected the interaction between GluN2B and STIM2.

### Removal of STIM1 or STIM2 does not affect total GluN1, GluN2A, or GluN2B protein content

**Fig. 6:**
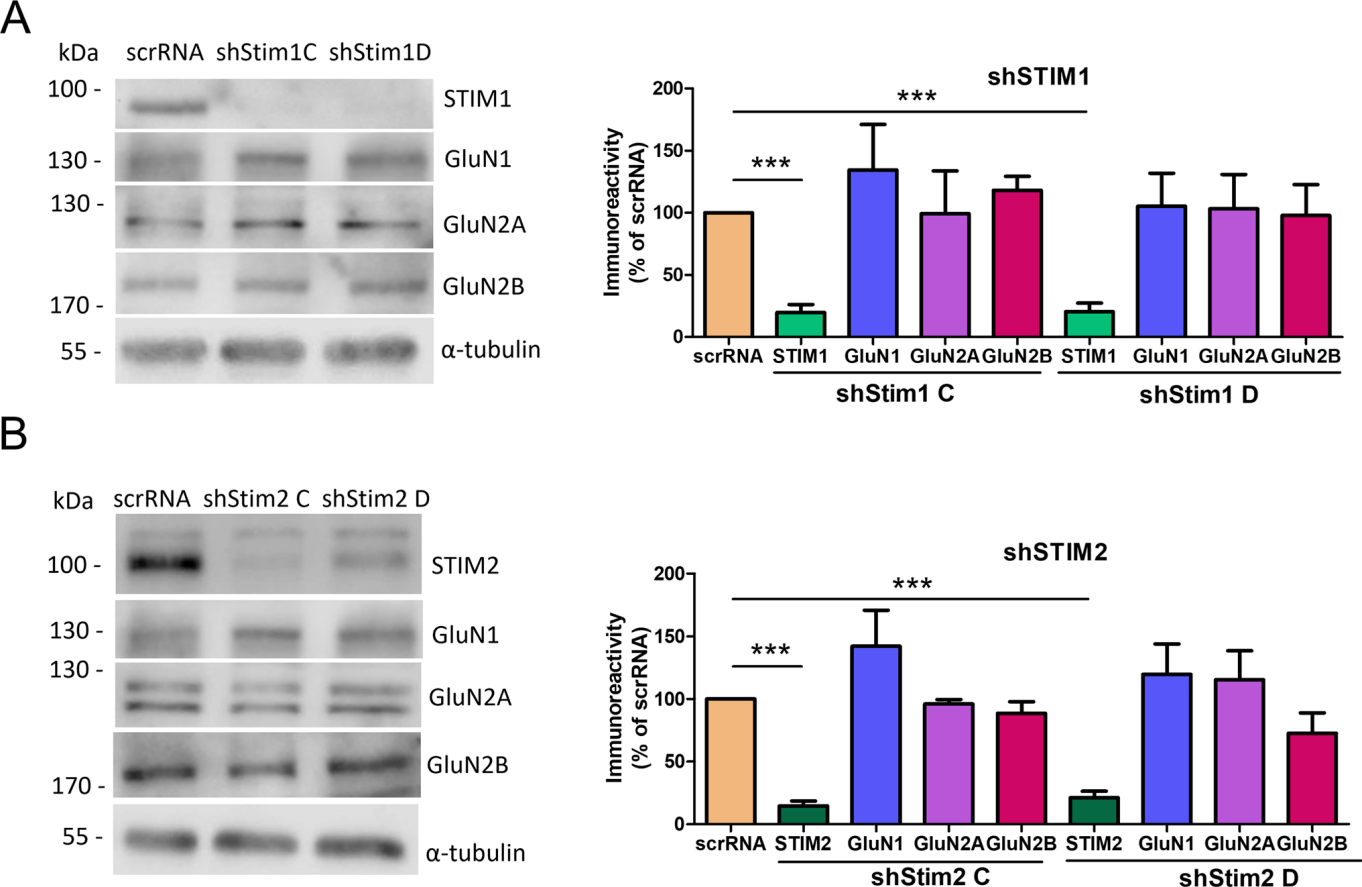
STIM1 and STIM2 knockdown does not influence the content of GluN1, GluN2A, or GluN2B in the cell lysate. **A**, **B** Representative Western blot and densitometric analysis of NMDAR subunits and STIM proteins in cell lysates of STIM1- and STIM2-silenced neurons (shStim1 C, shStim1 D, shStim2 C, and shStim2 D) and scrRNA. Total protein levels, determined by Ponceau S staining (see "Materials and methods" section), and tubulin were used as loading controls. The data were normalized to Ponceau S and are expressed as the mean ± SEM of three-to-five independent experiments. ***p < 0.001 (1-way Anova)

We next investigated the effect of STIMs on the content of NMDAR subunits. STIM proteins were knocked down with lentiviruses that carried a shRNA sequence against STIM1 (shStim1 C, shStim1 D) or STIM2 (shStim2 C, shStim2 D). Both STIM proteins were silenced specifically and effectively. shStim1 C and shStim1 D reduced STIM1 (but not STIM2; Fig. S1A) protein levels by an average of 80%, and shStim2 C and shStim2 D reduced STIM2 (but not STIM1; Fig. S1A) protein levels by 85% and 79%, respectively (Fig. 6A, B, Fig. S1B). To test the effect of lentiviral transduction and STIM protein silencing on neuronal cell viability, lactate dehydrogenase release was measured in wild-type, scrRNA, and shStim-transduced neurons, and no differences were observed (Fig. S6). Furthermore, none of the vectors altered the NMDAR protein level (Fig. 6A, B).

### STIM2 regulates NMDAR-mediated currents

**Fig. 7:**
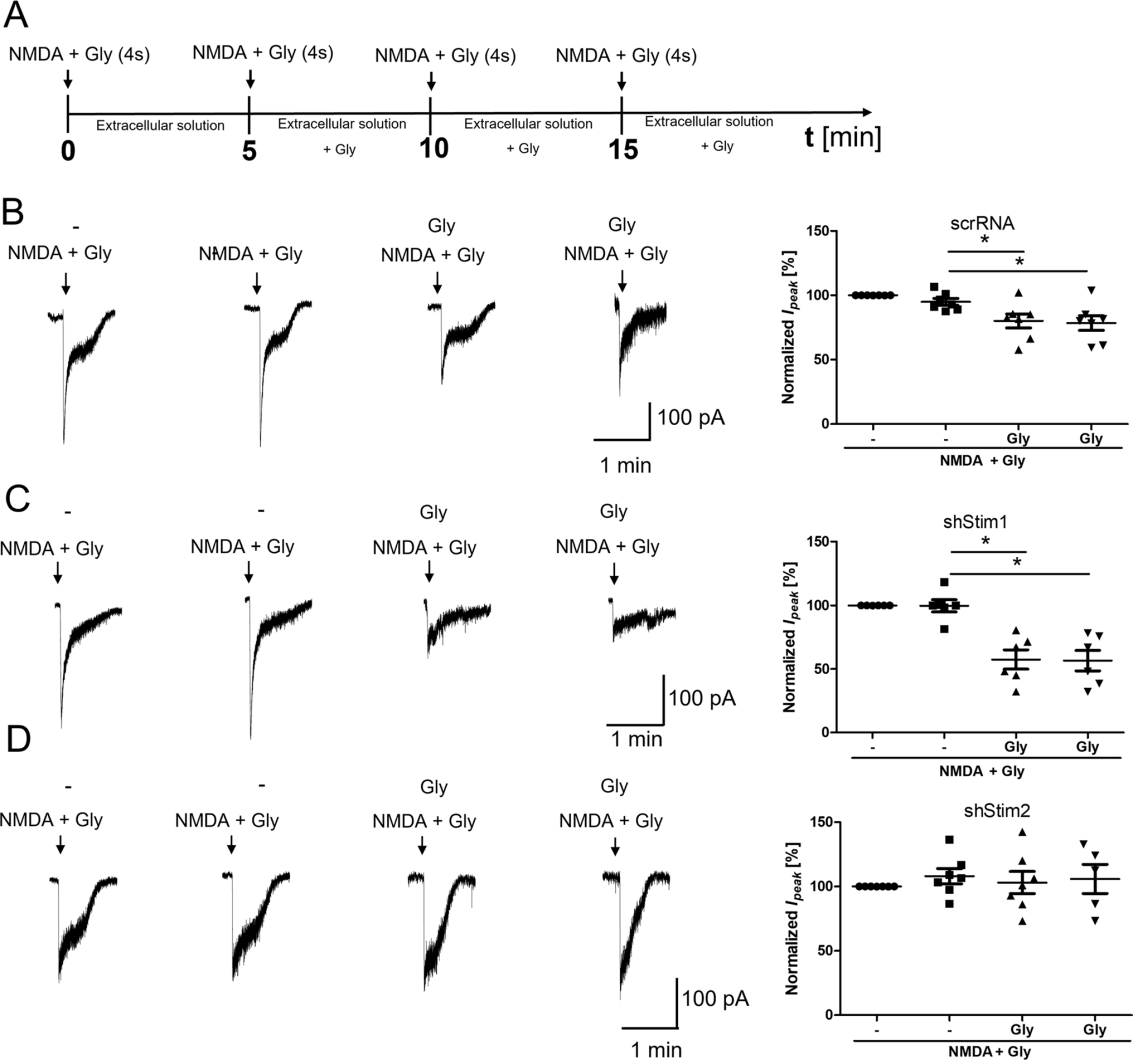
STIM2 knockdown suppresses the reduction of NMDAR-mediated currents following NMDAR overactivation.** A** Scheme of the experimental protocol. **B**–**D** Traces show examples of NMDAR currents that were recorded using protocols from one cell at a time. The plots show normalized NMDAR peak currents that were recorded in scrRNA-, shStim1 D-, and shStim2 D-transduced neurons that were treated 4 times with 50 µM NMDA and 100 µM glycine (NMDA+Gly) for 4 s. Peak currents were normalized to the first response that was evoked by the application of 50 µM NMDA and 100 µM glycine. The data are expressed as the mean ± SEM of three independent experiments. ***p < 0.001 (unpaired *t* test). *n* = 6–7 cells

To investigate functional effects of STIMs, NMDAR-mediated currents were recorded in dissociated cortical neurons following the knockdown of STIM1 or STIM2. Consistent with the results from wild-type neurons (Fig. 4B), short-term NMDAR overactivation in scrRNA neurons resulted in a statistically significant reduction of the NMDAR-mediated current amplitude to 70.49% ± 7.31% of the basal magnitude (Fig. 7B), although this change is less than in wild-type neurons. These results may suggest the prevention of NMDAR endocytosis by the lentivirus. Thus, the introduction of lentivirus into neurons is undoubtedly a kind of interference in neurons, which may affect the results of research, especially since cells with viruses are less sensitive to stimuli. For this reason, the results for neurons in the presence of scrRNA do not completely coincide with the results for wild-type neurons. However, to exclude the influence of the virus, in this (and subsequent) experiments, we compared the effects of NMDAR overactivation in cells treated with scrRNA-containing lentivirus with the response of cells after STIM protein silencing using the same viral vector.

Most importantly, after STIM2 silencing, the amplitude of NMDAR-mediated currents did not change (Fig. 7D). Short-term NMDAR overactivation in STIM1-silenced neurons decreased the NMDAR-mediated current amplitude to 57.47% ± 7.55% (Fig. 7C), which was similar to wild-type and scrRNA-transduced neurons (Figs. 4B, 7B). These results suggest that STIM2 but not STIM1 may regulate NMDAR-mediated currents via an influence on NMDAR endocytosis.

### Removal of STIM2 but not STIM1 inhibits GluN1, GluN2A, and GluN2B endocytosis after short-term NMDAR overactivation

Next, we tested whether the level of surface NMDARs was altered in STIM1- and STIM2-knockdown neurons after short-term NMDAR overactivation. Surface NMDARs were labeled with antibodies against their N-terminal extracellular epitopes under nonpermeable conditions, and the signal was detected with a surface protein quantification assay ELISA at an absorbance wavelength of 450 nm (see "Materials and methods" section).

**Fig. 8:**
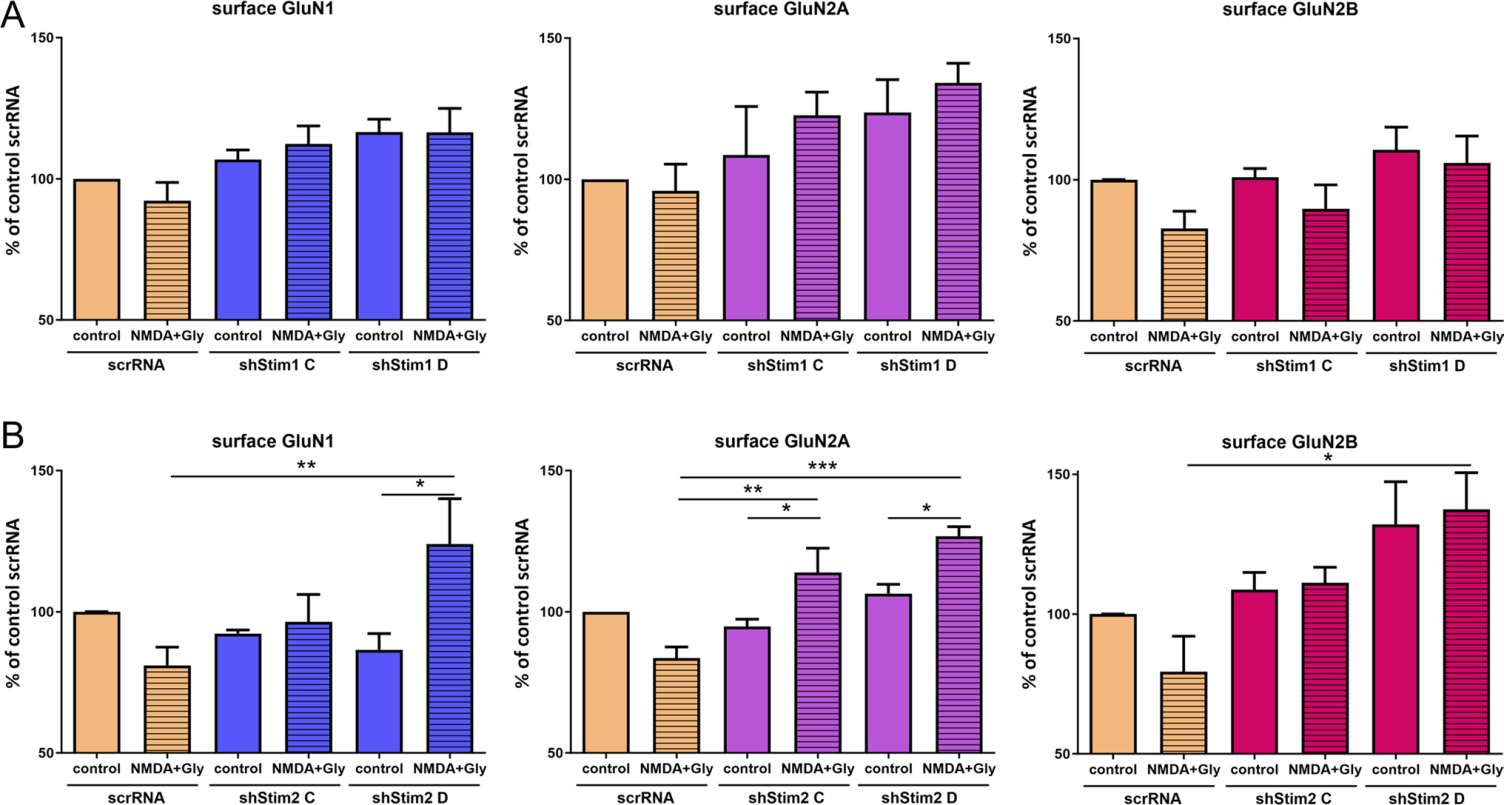
STIM2 knockdown inhibits the internalization of GluN1, GluN2A, and GluN2B from the PM after short-term NMDAR overactivation. **A**, **B** Expression levels of NMDAR subunits on the cell surface of neurons that were transduced with scrRNA, shStim1 (shStim1 C, shStim1 D), and shStim2 (shStim2 C, shStim2 D), expressed as a percentage of the control scrRNA. Surface GluN1, GluN2A, and GluN2B were detected by ELISA using antibodies that recognized their extracellular epitopes. The data are expressed as the mean ± SEM of three to seven independent experiments. *p < 0.05, **p < 0.01 (two-way Anova)

As shown in Fig. 8B, NMDAR overactivation in scrRNA neurons tends to reduce surface levels of GluN1, GluN2A, and GluN2B to 81.01% ± 6.43%, 83.61% ± 3.88%, and 79.36% ± 12.70%, respectively, which may suggest NMDAR internalization. In contrast, in STIM2-silenced neurons, surface levels of NMDARs did not decrease after excessive NMDAR activation (shStim2 C) and even slightly increased (shStim2 D) to 124.0% ± 15.99%, 126.8% ± 3.38%, and 137.5% ± 13.09%, respectively (Fig. 8B). STIM1 silencing did not statistically significantly influence surface levels of NMDARs after their overactivation (Fig. 8A). This may indicate that STIM2 but not STIM1 silencing inhibits NMDAR internalization from the PM after NMDAR overactivation.

**Fig. 9:**
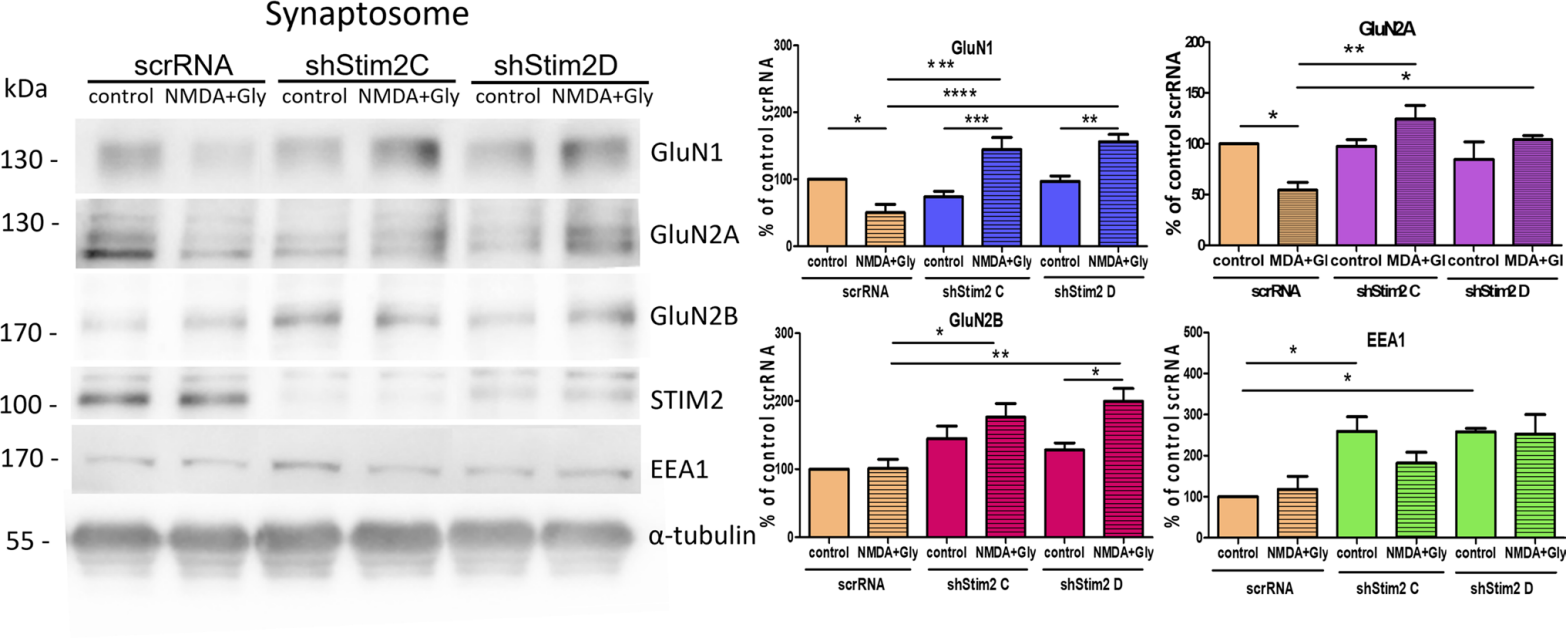
STIM2 knockdown inhibits the internalization of GluN1, GluN2A, and GluN2B from the synaptosome after short-term NMDAR overactivation. Representative Western blots and quantification of levels of NMDAR subunits and EEA1 in the synaptosome of neurons that were transduced with scrRNA, shStim2 C, or shStim2 D, expressed as a percentage of control scrRNA. Total protein levels, determined by Ponceau S staining (see "Materials and methods" section), and tubulin were used as loading controls. The data were normalized to Ponceau S and are expressed as the mean ± SEM of three-to-four independent experiments. *p < 0.05, **p < 0.01, ***p < 0.001 (2-way Anova)

To determine whether the difference between STIM1 and STIM2 is related to synaptic or extrasynaptic NMDARs, the effect of STIM knockdown on the amount of synaptosomal NMDARs was further investigated. Consistent with the results from wild-type neurons (Fig. 2), the NMDAR overactivation of scrRNA-transduced ones resulted in a significant reduction of synaptosomal GluN1 and GluN2A levels to 53.43% ± 12.19% and 54.38% ± 7.76%, respectively (Fig. 9), whereas the amount of GluN2B remained unaltered. STIM2 silencing inhibited post-activation NMDAR internalization in the synaptosome (Fig. 9) and significantly increased levels of synaptosomal GluN1, GluN2A, and GluN2B to 144.70% ± 17.82%, 124.90% ± 14.53%, and 176.36% ± 20.34% (shStim2 C) and 156.20% ± 10.90% 104.22% ± 2.69%, and 199.67% ± 22.96% (shStim2 D), respectively. STIM1 silencing did not alter synaptosomal NMDAR levels (Fig. S7). These results suggest that the silencing of STIM2 but not STIM1 inhibits NMDAR internalization from synaptic sites.

**Fig. 10:**
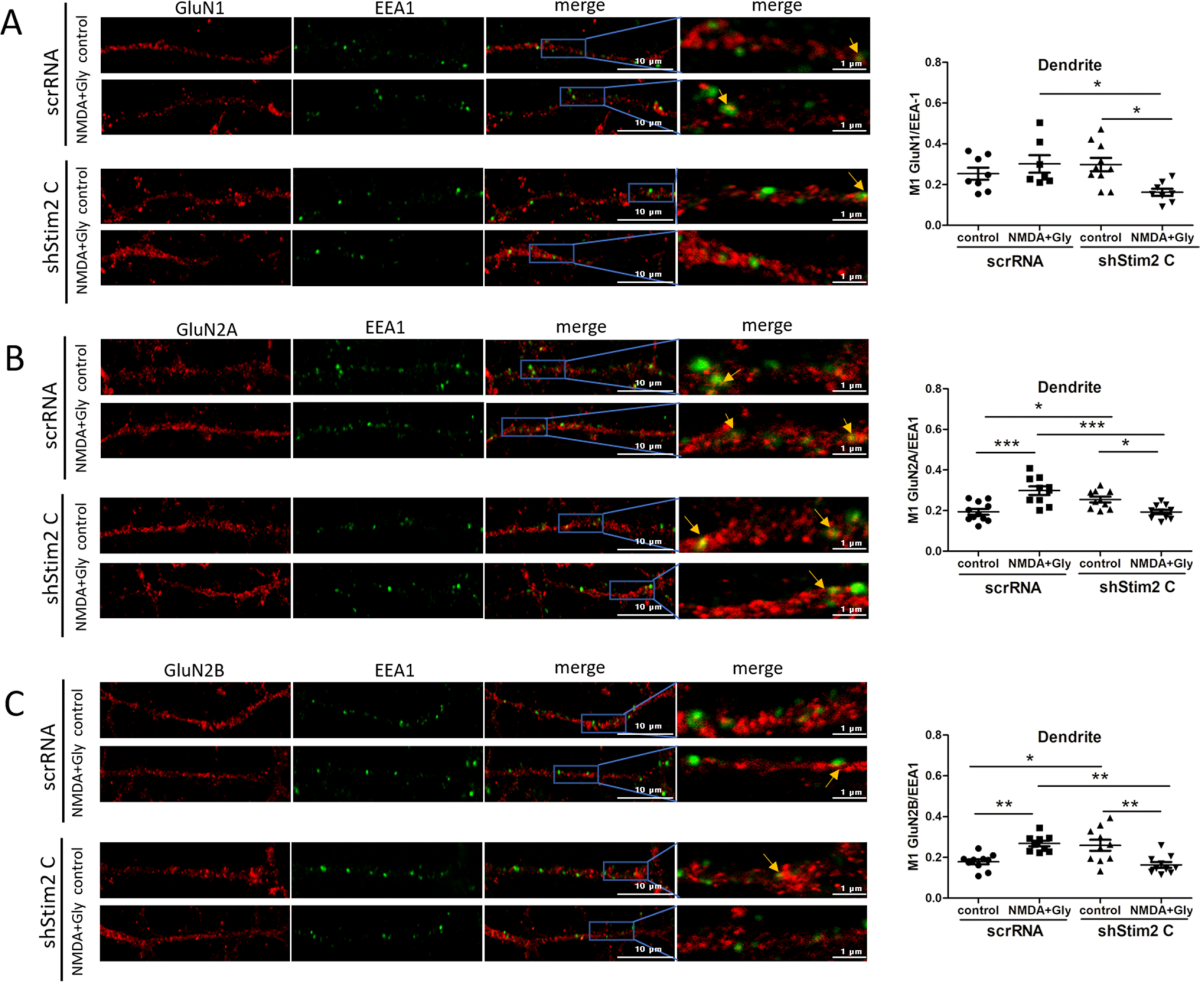
STIM2 knockdown decreases the co-localization of GluN1, GluN2A, and GluN2B with EEA1, a marker of early endosomes, in dendrites after short-term NMDAR overactivation. Representative deconvoluted confocal microscopy images of NMDAR subunits and EEA1 in dendrites from neurons that were transduced with scrRNA or shStim2 C and quantification of co-localization points using Manders’ co-localization coefficient (M1). **A**–**C** The co-immunostaining of GluN1, GluN2A, and GluN2B (red) with EEA1 (green) is shown. The “merge” columns show the labeling of NMDAR subunits overlapped with EEA1 labeling. All images were taken from a single optical section. Scale bar = 10 µm and 1 µm. The data are expressed as the mean ± SEM of three independent experiments. *p < 0.05, **p < 0.01, ***p < 0.001 (1-way Anova)

We also found that STIM2 silencing increased the amount of EEA1 in the synaptosome (Fig. 9). This raised the question of whether STIM2 knockdown influences NMDAR endocytosis after NMDAR overactivation. Thus, GluN1, GluN2A, and GluN2B were immunofluorescently stained with EEA1 (Fig. 10). In scrRNA neurons, immunostaining was consistent with the results from wild-type neurons (Fig. 3). Short-term NMDAR overactivation increased the co-localization of GluN1, GluN2A, and GluN2B with EEA1 in both the cell body (Fig. S8) and dendrites (Fig. 10), indicating an increase in NMDAR endocytosis. Although STIM2 knockdown and NMDAR overactivation did not affect the co-localization of NMDAR subunits with EEA1 in the cell body (Fig. S8), they reduced co-localization in dendrites (Fig. 10). Consistent with the data in Figs. 8A and S7, STIM1 silencing did not affect the co-localization of NMDAR subunits and EEA1 in either the cell body (Fig. S8) or dendrites (Fig. S9). Altogether, these results suggest that the silencing of STIM2 but not STIM1 inhibits the endocytosis of GluN2A- and GluN2B-containing NMDARs in dendrites.

## Discussion

**Fig. 11:**
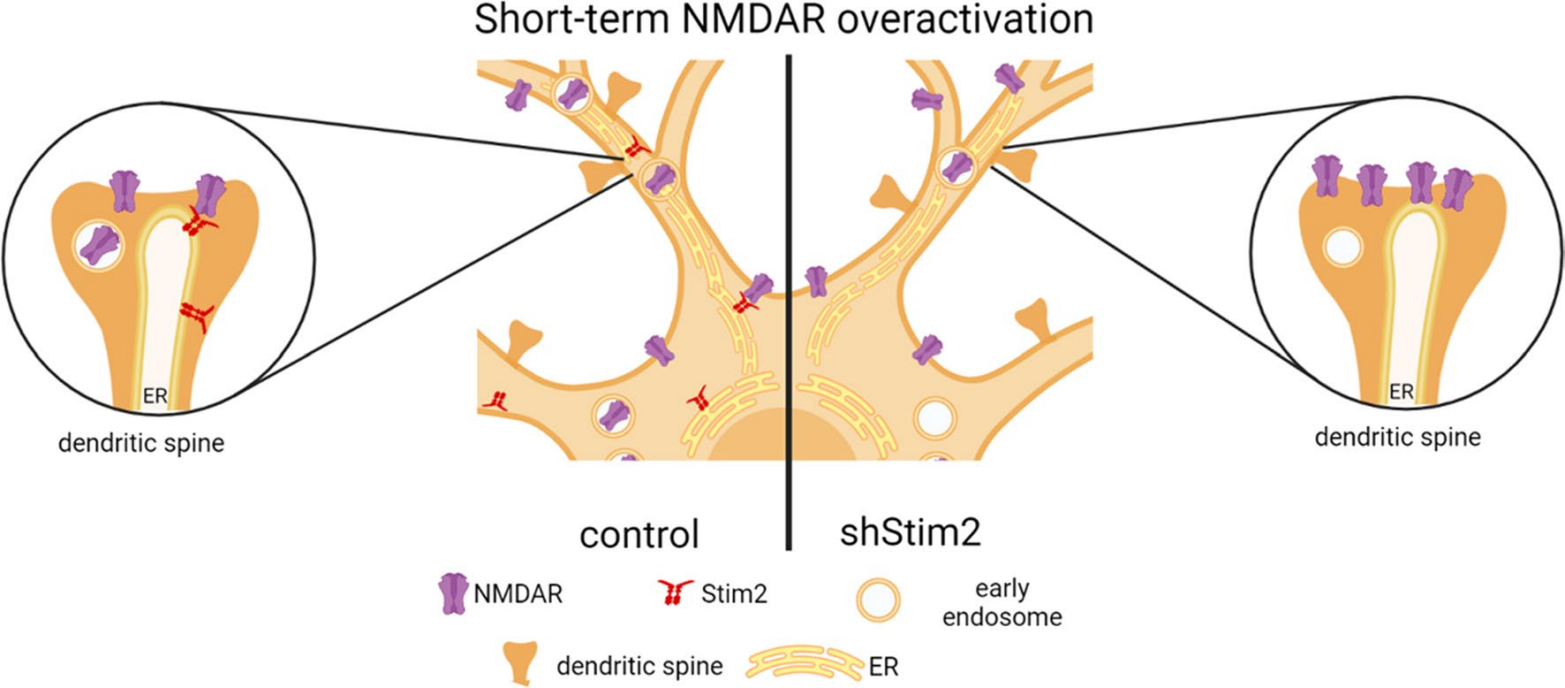
Proposed role of STIM2 in NMDAR endocytosis in rat cortical neurons. In wild-type neurons (control), short-term NMDAR overactivation causes the trafficking of GluN2 subunits of NMDARs from the plasma membrane (PM) of the cell body and dendrites to early endosomes. STIM2–NMDAR complexes are formed. In STIM2-knockdown (shStim2) neurons, NMDAR endocytosis that is induced by short-term NMDAR overactivation is significantly reduced, and receptors accumulate at the PM at synaptic and extrasynaptic sites

In this study, we found that STIM2, a known activator of SOC channels, reduced NMDA-evoked currents and the surface expression of NMDARs by binding directly to GluN2 subunits of NMDARs. We also found that after short-term NMDAR overactivation, STIM2 silencing inhibited the internalization of GluN1, GluN2A, and GluN2B in synapses and reduced the co-localization of NMDAR subunits with endosomes in dendrites. Altogether, our findings establish a previously unrecognized function of STIM2 protein that involves the promotion of NMDAR endocytosis after its short-term overactivation, thus shedding new light on the mechanism of NMDAR trafficking in cultured cortical neurons. The present results are summarized in Fig. 11.

Under physiological conditions, NMDAR activation results in ER Ca^2+^ store depletion and the initiation of SOCE [30, 34, 75, 76]. Baba et al. showed that SOCE inhibitors attenuate both Ca^2+^ influx through the NMDAR and long-term potentiation in hippocampal pyramidal cells [34], suggesting that synaptic NMDAR-activated SOCE is crucial for long-term potentiation. Moreover, the depolarization of hippocampal neurons with glutamate causes Ca^2+^ influx through NMDARs and L-type VGCCs to release Ca^2+^ from the ER and activate STIM1, which can further control the structural plasticity of dendritic spines [77]. As demonstrated in the present study, short-term NMDAR overactivation reduced STIM1 and NMDAR levels but not STIM2 levels in synaptosomes (Fig. 2), whereas the amount of STIM1 and STIM2 in total lysates and probably associated with the PM remaining unchanged (Fig. 1B). However, we did not observe STIM1–GluN2A and STIM1–GluN2B associations. These results suggest that after short-term NMDAR overactivation, STIM1 may migrate from synaptic to extrasynaptic sites. This hypothesis requires further research.

Using biochemical and electrophysiological methods, we found that in wild-type neurons NMDAR overactivation with high NMDA/glycine concentrations also led to the endocytosis of GluN2A- and GluN2B-containing NMDARs. The rate of their internalization was higher in the PM than in synaptosomes, suggesting that both synaptic and extrasynaptic NMDARs are primed for endocytosis. These results are supported by the previous studies that showed that synaptic and extrasynaptic NMDARs but no other ionotropic receptors (e.g., AMPARs) are internalized after high NMDA and glycine treatment [51]. This may in turn result in a reduction of Ca^2+^ influx that may exert a protective effect on neurons.

STIMs can act as modulators of Ca^2+^ signaling between cellular compartments [78–80], thereby supporting NMDAR trafficking both to and from the PM. The prediction from this hypothesis is that STIM and NMDARs may form a complex. Indeed, our Co-IP results showed a direct interaction between STIM2 and the NMDAR GluN2 subunits in resting neurons, which is consistent with earlier research [42]. Moreover, the formation of STIM2-NMDAR complexes appears to change after SOCE activation [42] and, as demonstrated here, after short-term NMDAR overactivation followed by receptor endocytosis. A novel finding in the present study was that the STIM2–NMDAR2B and STIM2–NMDAR2A interactions were enhanced after NMDAR endocytosis, increasing the likelihood that STIM2 is involved in the local transport of NMDARs. Short-term NMDAR overactivation induced the endocytosis of GluN2A- and GluN2B-containing NMDARs into early endosomes, but did not affect STIM protein levels associated with the cell surface (Figs. 1, 2, 3). Accordingly, we suggest that short-term NMDAR overactivation results in the formation of STIM2–GluN2B, and STIM2–GluN2A complexes in cytoplasm or the ER. We can speculate that this way STIM2 modulates NMDAR internalization, as its silencing significantly reduces endocytosis of this receptor.

The neuronal ER, where STIMs reside and NMDARs are synthesized [46–48], is mainly found in the cell body but can also enter dendrites and dendritic spines [14, 81, 82]. Therefore, an obvious site of interaction between STIMs and NMDARs may also be within these structures. Consistent with this possibility, the co-localization of STIM2 proteins with GluN2 was apparent in both the cell body and dendrites. However, upon overactivation of NMDARs, the co-localization of STIM2 proteins with GluN2 only increased in the cell body. In the present study, a population of STIM1 proteins was also found on the surface of neurons, which is consistent with the previous results [23–25, 27]. Keil et al. showed that many STIM1-GFP surface puncta mobilized into large punctate clusters that matched with PSD95 clusters, indicating that STIM1-GFP can be integrated into the PM in both somatic and dendritic compartments of hippocampal neurons [27]. The well-known 90-kD STIM1 (STIM1S) is the primary splice variant in the rodent brain. In addition, a 115-kD STIM1 named STIM1L that is associated with actin [83, 84] and a neuron-specific splice variant of 62-kD STIM1 named STIM1B that localizes to presynaptic ER have also been identified [31]. In our experiments, we also observe a ~ 60 kDa band of the short STIM1 isoform with a molecular weight similar to STIM1B (Fig. S1A) which was previously demonstrated in various brain regions [31]. Additionally, it was not silenced using STIM1 shRNA plasmids, as was the case with the conventional STIM1 isoform. Interestingly, compared to STIM1, STIM1B does not have 170 amino acids, but has an additional peptide of 26 amino acids in the cytoplasmic domain. Therefore, the shRNA sequence we used likely targets the C-terminal region of STIM1 within these 170 amino acids. Since STIM1B also mediates ICRAC reduction, it may also be responsible for reducing NMDAR-mediated current amplitude in neurons with shRNA against STIM1 more strongly than in neurons with scrRNA control (Fig. 7). This may be due to the lack of a microtubule tracking domain in STIM1B and, consequently, its slower transport toward the cell membrane [31]. Using the surface biotinylation and subcellular fractionation methods, we also detected a population of STIM2, in addition to STIM1, in PM of neurons. STIM2 contains a functional di-lysine ER retention signal KKXX in the K-rich domain, which is most likely responsible for STIM2 retention in the ER [85, 86]. However, there is also a STIM2 population—the so-called preSTIM2, which unlike STIM2 does not have an ER location. preSTIM2 stably binds to the plasma membrane, where it regulates the basal Ca^2+^ concentration in a storage-independent manner [87]. In our studies using surface protein biotinylation method, we do not observe increased MW on the blots for cell surface STIM2, which could exclude the presence of preSTIM2. Therefore, it is likely that STIM2 deposits in the PM between the lipid layers or simply associates with the PM while still in the ER rather than being localized to the PM. These studies require further follow-up studies. We show that STIMs were also present in synaptosomes, which is consistent with earlier studies of the hippocampus and striatum of adult rodent brains [18, 39, 71, 72] that STIM1 and particularly STIM2 proteins localize to dendritic spines and synapses and are present in synaptosomes [17, 32, 33, 39, 70–72]. Recently, it was shown that in hippocampal neurons, on average, ∼40% of the total STIM2 volume was in the axons and  ~ 30% in dendrites and synapses. In addition, approximately 60% of the PSD95, post-synaptic marker, co-localized with STIM2 [32]. 30–45% of mushroom dendritic spines alone were labeled with STIM1 and STIM2 proteins [17]. These levels of STIMs in the post-synaptic part of spines are consistent with the previous reports showing that approximately 10–50% of dendritic spines of neurons contain a smooth ER [73, 88–91]. Interestingly, the ER in the dendritic spines of the rat hippocampus occupied 12–40% of the spine area [73]. In turn, Pchitskaya et al. observe the same amount of STIM2 protein in both lysates and synaptosomes [71]. Garcia-Alvarez et al. show STIM2 enrichment in synaptosomes and even greater enrichment in post-synaptic density [39]. In addition, Orai2 and Orai1 can pull down STIM2 from synaptosomal lysates [18]. In the present study, STIM2-NMDAR co-localization did not change in dendrites but increased in the cell body after short-term NMDAR overactivation.

As only STIM2 and GluN2 increased their interactions with each other upon NMDAR overactivation and appeared to co-localize, we further investigated whether STIM2 compared to STIM1 alters the expression of receptors on the cell surface and in synaptosomes. Our data indicated that under control conditions, neither STIM1 nor STIM2 influenced the level of any NMDAR subunits in the PM. Similarly, after STIM2 silencing, the GluN2A–PSD95 associations remained unaltered [39]. Furthermore, STIM2 knockdown increased the amount of EEA1 in synaptosomes and its co-localizations with GluN2 in dendrites (Figs. 9, 10), which was not observed after the silencing of STIM1 (Figs. S8, S9). More importantly, upon short-term NMDAR overactivation, STIM2 silencing prevented the loss of surface and synaptosomal GluN2B as well as GluN1 and GluN2A and reduced their co-localization with EEA1 in dendrites. Moreover, whole-cell patch-clamp recording in cortical neurons clearly showed that, compared to scrRNA, the knockdown of STIM2 but not STIM1 increased the magnitude of NMDA- and glycine-induced currents, suggesting that the number of NMDARs on the surface of neurons is regulated by STIM2. These results support our biochemical analyses and highlight the physiological significance of our results. Collectively, our data provide evidence of the ability of STIM2 to induce NMDAR endocytosis under such conditions. These findings correlate well with the previous data that showed an increase in Ca^2+^ influx through the NMDAR after STIM2 silencing and a decrease after transient STIM2 overexpression [42]. In turn, STIM1 protein after NMDAR activation appears to migrate from synaptic to extrasynaptic sites, and its pivotal role remains the regulation of ER Ca^2+^ levels in the mechanism of SOCE. These results suggest that STIM2 protein might be a regulator of ionotropic receptor internalization. Previous studies showed that STIM2 interacts with AMPAR subunits and is involved in AMPAR internalization in neurons [39–41], thus providing some evidence to support our hypothesis.

Our results may have far-reaching functional consequences when considering the crucial role of NMDARs in brain physiology (e.g., neuronal development and synaptic plasticity) and pathology (e.g., excitotoxicity). The overactivation of GluN2-containing NMDARs and impairments in the endocytosis of these receptors are critical for NMDA-induced excitotoxicity and may contribute to the development of traumatic brain injury and neurodegenerative diseases, such as Alzheimer’s disease and Huntington’s disease [49, 92–98]. During ischemic stroke, GluN2B–NMDAR endocytosis is inhibited, which, together with excessive activation of the receptor, contributes to the damage and death of neuronal cells [98]. The glycine-induced internalization of NMDARs provides neuroprotection in this case [50]. The dysfunction of STIM proteins, particularly STIM2, has also been detected in various neuronal cell and animal models of neurological diseases [11, 28, 49, 99–103]. Further studies of the relationship between STIM2 and NMDAR endocytosis may reveal new therapeutic options for various disorders of the central nervous system.

In conclusion, the present study broadens our understanding of the relationship between NMDARs and STIM proteins and confirms the existence of a novel function of STIM2 protein in rat cortical neurons. We postulate that a novel function of STIM2 is its ability to regulate NMDAR endocytosis after excessive NMDAR activation through a direct interaction with these receptors. After NMDAR overactivation, STIM2 forms complexes with NMDAR subunits in the synaptic ER, possibly thereby modulating their transport into the cell.

### Supplementary Information

Below is the link to the electronic supplementary material.

Supplementary file1 (PDF 1544 KB)

## Data Availability

The authors declare that data supporting the results of this study are available in the article and its supplementary materials. Further resource information is available from the corresponding author upon reasonable request.
