# Supplementary material for: STIM2 regulates NMDA receptor endocytosis that is induced by short-term NMDA receptor overactivation in cortical neurons
Source: Cell Mol Life Sci. 2023 Nov 21;80(12):368. doi: 10.1007/s00018-023-05028-8 (PMC10663207; doi:10.1007/s00018-023-05028-8)
Supplement: Supplementary file 1 — Supplementary file1 (PDF 1544 KB) [file 18_2023_5028_MOESM1_ESM.pdf]

Supplementary Information

Cellular and Molecular Life Sciences

STIM2 regulates NMDA receptor endocytosis that is induced by short-term NMDA receptor overactivation in cortical neurons

Karolina Serwach[1], Ewa Nurowska[2], Marta Klukowska[1], Barbara Zablocka[1], Joanna Gruszczynska-Biegala[1, *]

[1] Molecular Biology Unit, Mossakowski Medical Research Institute, Polish Academy of Sciences, Warsaw, Poland; [2] Chair and Department of Pharmacotherapy and Pharmaceutical Care, Centre for Preclinical Research and Technology (CePT), Medical University of Warsaw, Poland

KS: ORCID 0000-0002-1836-3761
EN: ORCID 0000-0002-3566-6465
BZ: ORCID 0000-0003-3116-7295
JG-B: ORCID 0000-0003-3541-2550

*Corresponding author
J. Gruszczynska-Biegala: jgruszczynska@imdik.pan.pl

Fig. S1

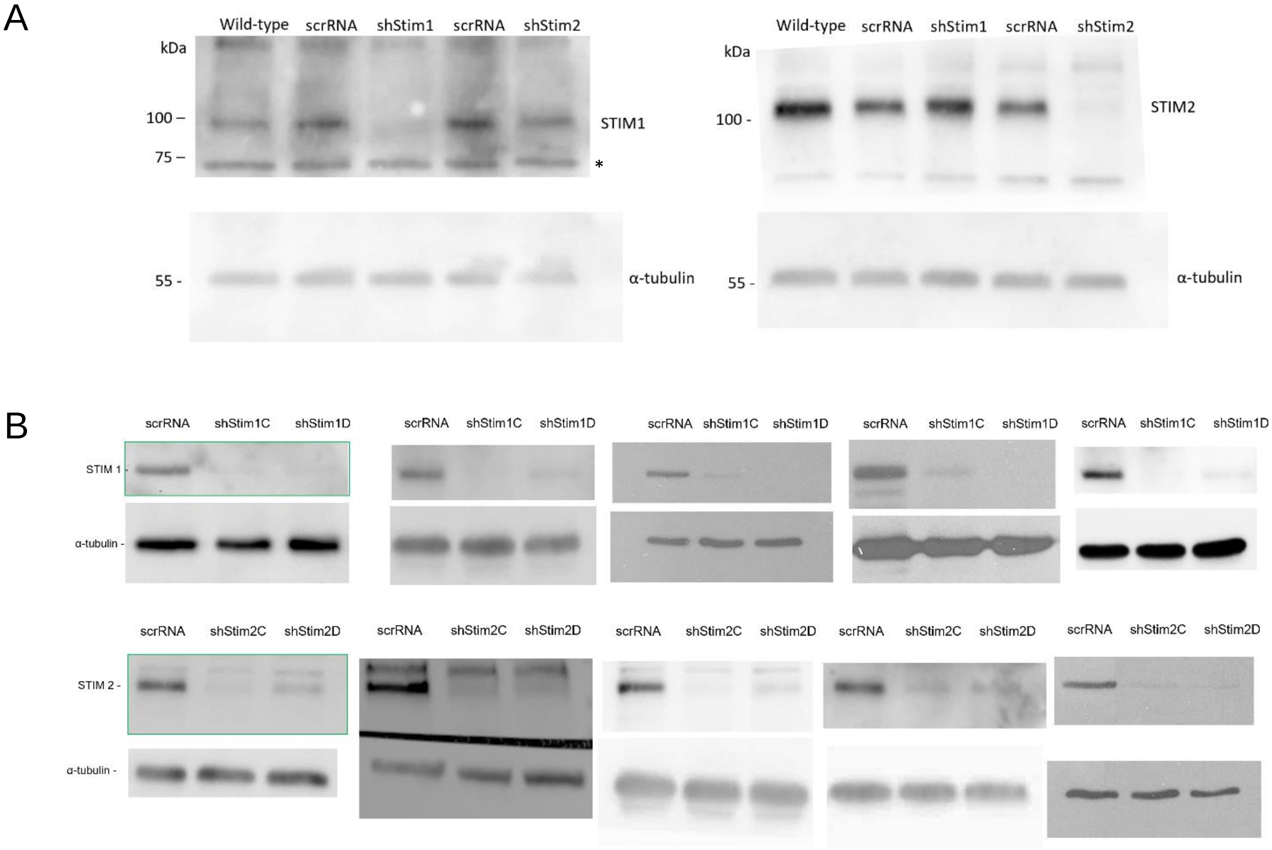

Figure S1. Knockdown of STIM1 or STIM2 reduces expression of STIM1 or STIM2, respectively, but not other proteins. (**A**) Wild-type cortical neurons or transduced with control scrRNA, shStim1 or shStim2 were immunoblotted and probed for STIM1, STIM2 and tubulin. The asterisk (*) indicates an additional ~ 62-kDa band, likely representing STIM1B. (**B**) Western Blots of proteins immunoblotted and probed for STIM1 and STIM2 that were taken for densitometric analysis in Figure 6.

Figure S2

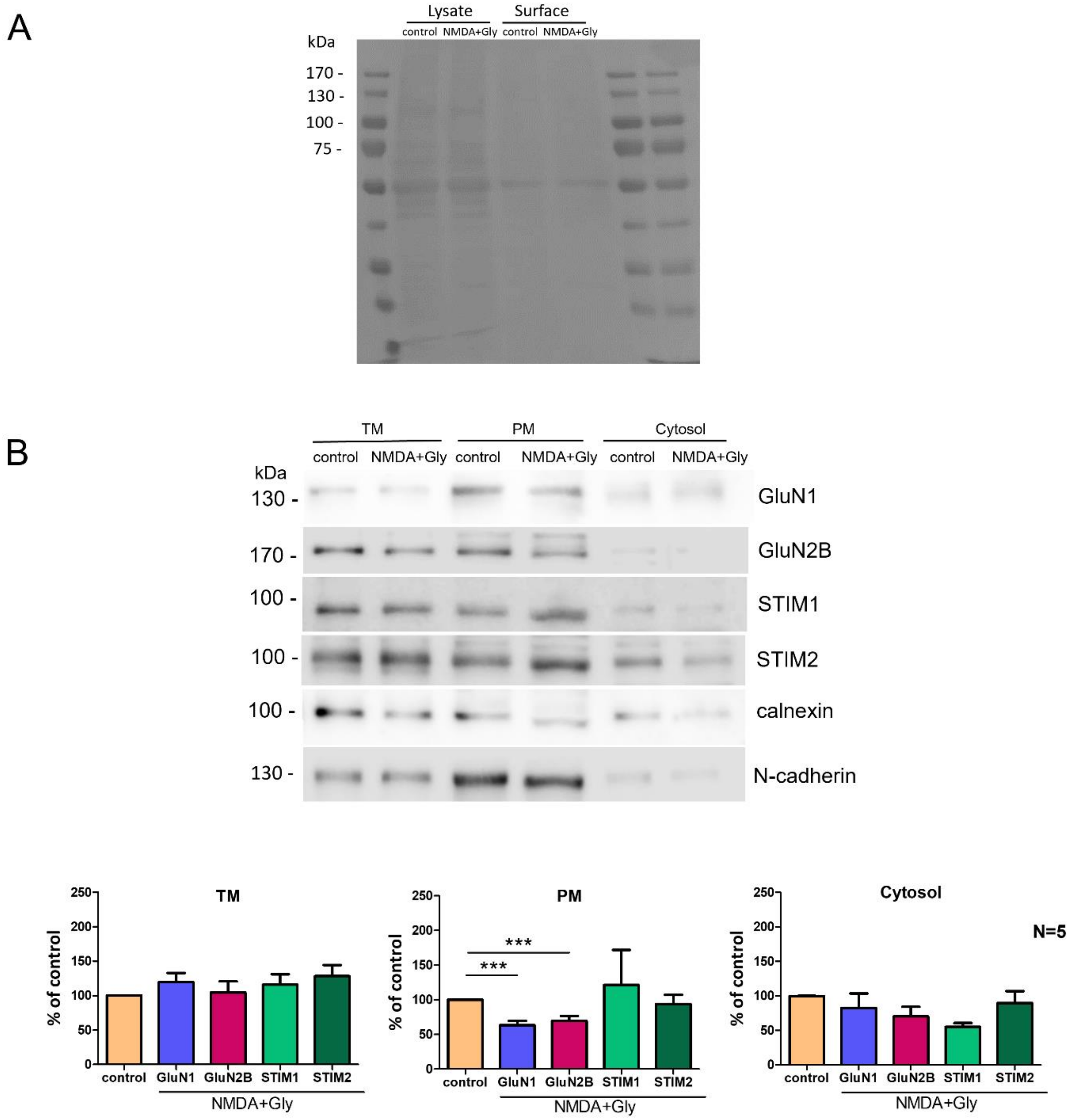

Figure S2. Expression of STIM proteins in subcellular neuronal fractions. (**A**) Ponceau S staining of the membrane shown in Figure 1. (**B**) Wild-type cortical neurons were subjected to subcellular fractionation into total membrane (TM), plasma membrane (PM) and cytosolic (Cytosol) fractions. Proteins were immunoblotted and probed for GluN1, GluN2B, STIM1, STIM2, calnexin (ER membrane) and N-cadherin (PM). The data are expressed as the mean ± SEM of four to five independent experiments.***$p < 0.001$ (unpaired t-test).

Fig. S3

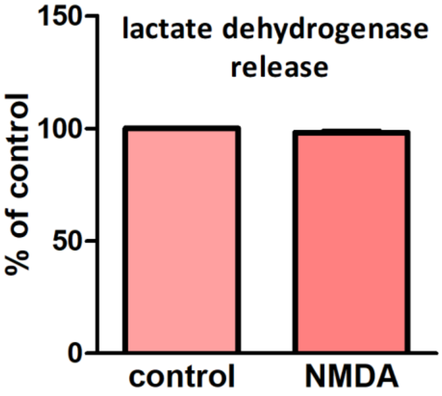

Figure S3. Short-term NMDAR overactivation does not influence neuronal cell survival *in vitro*. Lactate dehydrogenase release was measured in control neurons and NMDA- and glycine-treated neurons for 15 min. The data are expressed as the mean ± SEM of four independent experiments.

Fig. S4

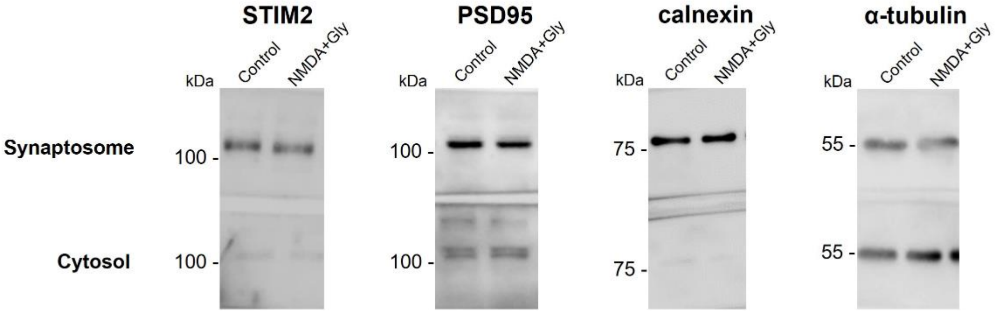

Figure S4. Expression of STIM2 in synaptosome. Wild-type cortical neurons were subjected to fractionation into synaptosomal (Synaptosome) and cytosolic (Cytosol) fractions. Proteins were immunoblotted and probed for STIM2, PSD95 (synaptosome), calnexin (ER membrane) and tubulin (cytosol).

Figure S5

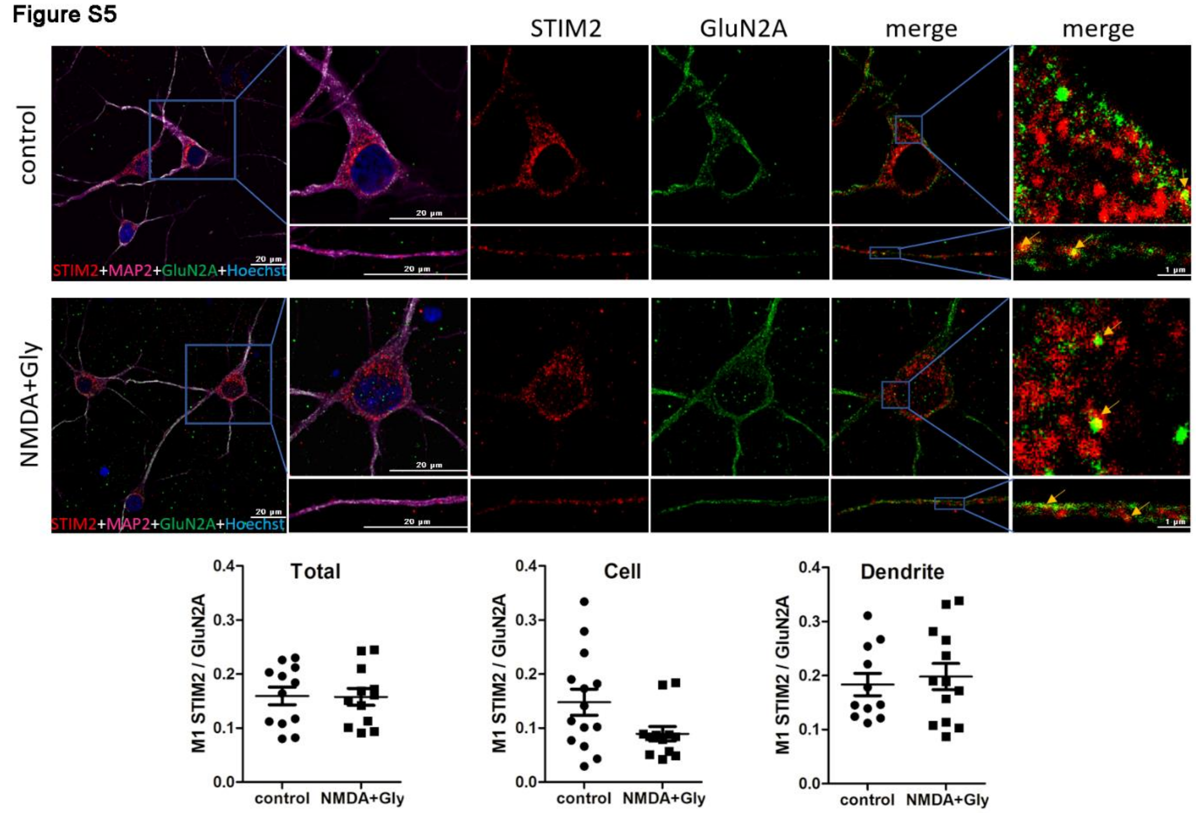

Figure S5. Short-term NMDAR overactivation decreases STIM1 co-localization with GluN2B and does not influence the co-localization of STIM2 with GluN2A in cortical neurons *in vitro*. (**A**, **C**) Representative confocal microscopy images of STIM1 with GluN2B and STIM2 with GluN2A. The co-immunostaining of STIM1/STIM2 (red), GluN2B/GluN2A (green), MAP2 (magenta), and nuclei (blue) is shown. The "merge" column shows STIM1 labeling overlapped with GluN2B labeling and STIM2 labeling overlapped with GluN2A labeling, and the last column shows their co-localization. All images were taken from a single optical section in the middle of the cell. Scale bar = 20 µm. (**B, D**) The Manders' co-localization coefficient (M1) was calculated to quantify the co-localization of STIM1 with GluN2B and STIM2 with GluN2A in the entire field of view (Total), single cell (Cell), and dendrites (Dendrite). The data are expressed as the mean ± SEM of three independent experiments (unpaired t-test).

Fig. S6

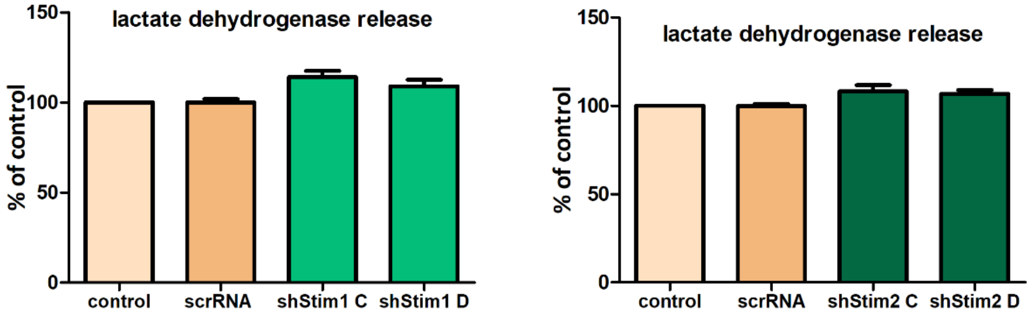

Figure S6. Lentiviral transduction of cortical neurons *in vitro* with scrRNA, shStim1, and shStim2 does not influence their survival. Lactate dehydrogenase release was measured in wildtype neurons and neurons that were transduced with scrRNA or two different shRNA constructs that induce the knockdown of STIM1 (**A**) and STIM2 (**B**). The data are expressed as the mean ± SEM of five to six independent experiments.

Fig. S7

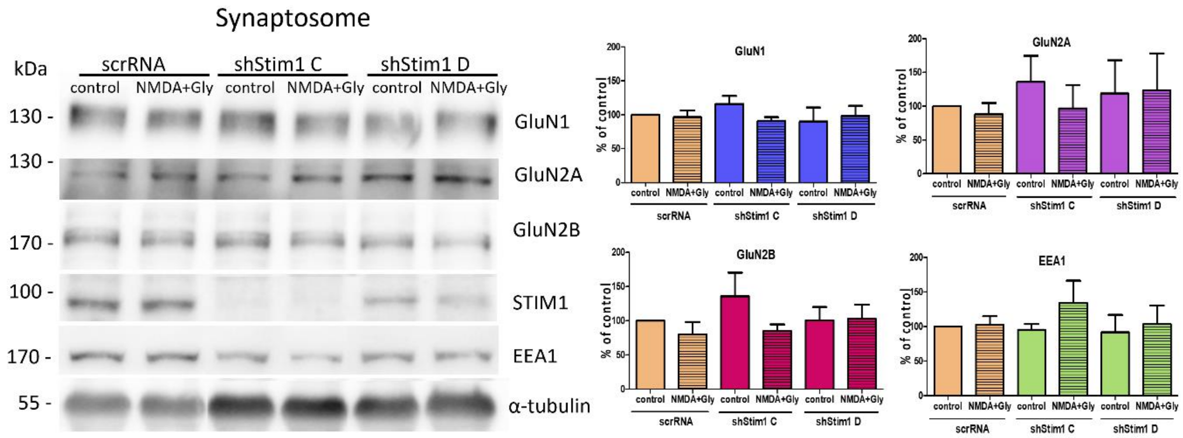

Figure S7. STIM1 knockdown does not influence the internalization of GluN1, GluN2A, or GluN2B from the synaptosome after short-term NMDAR overactivation in cortical neurons *in vitro*. Representative Western blots and quantification of the number of NMDAR subunits and EEA1 in the synaptosome of neurons that were transduced with scrRNA, shStim1 C, or shStim1 D, expressed as a percentage of control untreated scrRNA, are shown. Total protein levels, determined by Ponceau S staining, were used as loading controls. The data are expressed as the mean ± SEM of three to five independent experiments and were analyzed using Anova.

Fig. S8

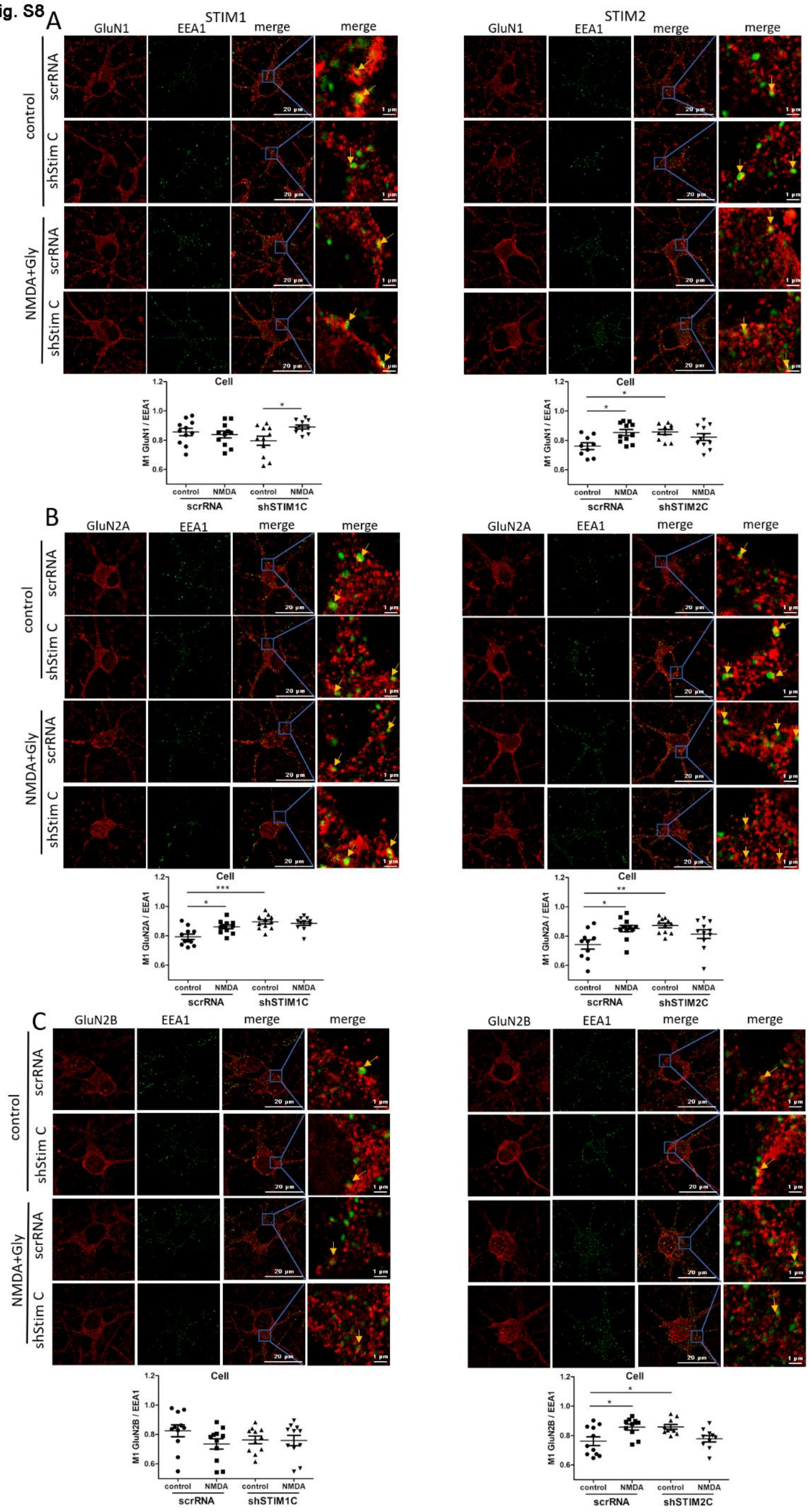
A
STIM1
GluN1
EEA1
merge
merge
control
scrRNA
shStim C
NMDA+Gly
20 μm
1 μm
Cell
M1 GluN1 / EEA1
control
NMDA
scrRNA
shSTIM1C
STIM2
shSTIM2C
B
GluN2A
M1 GluN2A / EEA1
C
GluN2B
M1 GluN2B / EEA1

Figure S8. Neither STIM1 nor STIM2 knockdown influence the co-localization of GluN1, GluN2A, or GluN2B with EEA1, a marker of early endosomes, in the cell body following short-term NMDAR overactivation. (**A**, **C**, **E**) Representative confocal microscopy images of cortical neurons transduced *in vitro* with scrRNA, shStim1 C, or shStim2 C. The co-immunostaining of GluN1, GluN2A, and GluN2B (red) and EEA1 (green) is shown. The "merge" column shows GluN1/GluN2A/GluN2B labeling overlapped with EEA1 labeling, and the last column shows their co-localization. All images were taken from a single optical section in the middle of the cell. Scale bar = 20 μm. (**B**, **D**, **F**) Quantification of co-localization points using Manders' co-localization coefficient (M1). The data are expressed as the mean ± SEM of three independent experiments. $^*p < 0.05$, $^{**}p < 0.01$, $^{***}p < 0.001$ (1-way Anova).

Fig. S9

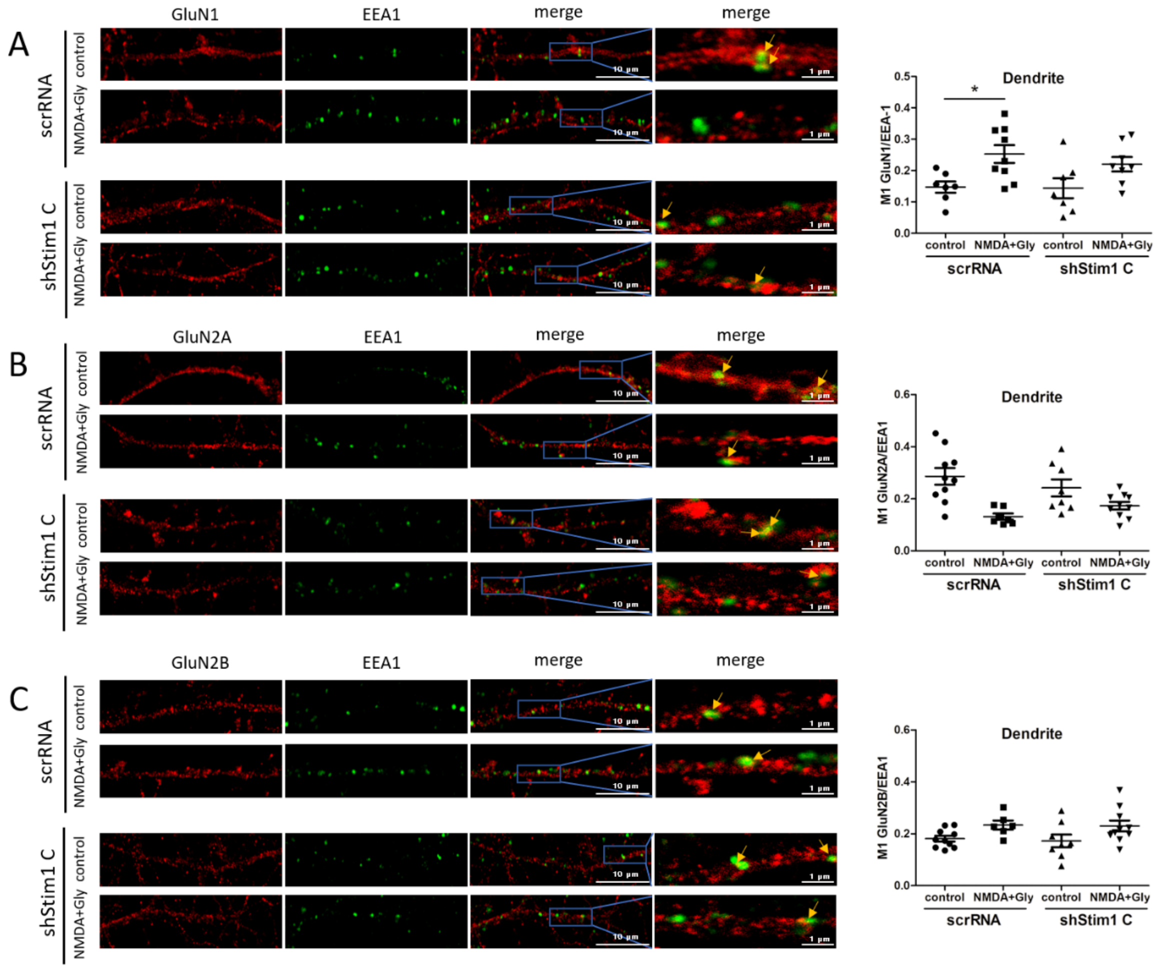

Figure S9. STIM1 knockdown does not influence the co-localization of GluN1, GluN2A, or GluN2B with EEA1, a marker of early endosomes, in dendrites after short-term NMDAR overactivation in cortical neurons *in vitro*. (**A, C, E**) Representative confocal microscopy images of dendrites that were derived from neurons that were transduced with scrRNA or shStim1 C. The co-immunostaining of GluN1, GluN2A, and GluN2B (red) and EEA1 (green) is shown. The "merge" column shows GluN1/GluN2A/GluN2B labeling overlapped with EEA1 labeling, and the last column shows their co-localization. All images were taken from a single optical section. Scale bar = 20 µm. (**B, D, F**) Quantification of co-localization points using Manders' co-localization coefficient (M1). The data are expressed as the mean ± SEM of three independent experiments. $*p < 0.05$ (1-way Anova).
